# High‐Performance n‐Type Organic Thermoelectrics Enabled by Synergistically Achieving High Electron Mobility and Doping Efficiency

**DOI:** 10.1002/advs.202302629

**Published:** 2023-08-08

**Authors:** Kui Feng, Junwei Wang, Sang Young Jeong, Wanli Yang, Jianfeng Li, Han Young Woo, Xugang Guo

**Affiliations:** ^1^ Department of Materials Science and Engineering Southern University of Science and Technology Shenzhen Guangdong 518055 China; ^2^ Academy for Advanced Interdisciplinary Studies Southern University of Science and Technology Shenzhen Guangdong 518055 China; ^3^ Department of Chemistry Korea University Anamro 145 Seoul 02841 Republic of Korea

**Keywords:** electrical conductivity, electron mobility, n‐doping efficiency, organic thermoelectrics, side‐chain engineering

## Abstract

n‐Doped polymers with high electrical conductivity (*σ*) are still very scarce in organic thermoelectrics (OTEs), which limits the development of efficient organic thermoelectric generators. A series of fused bithiophene imide dimer‐based polymers, PO8, PO12, and PO16, incorporating distinct oligo(ethylene glycol) side‐chain to optimize *σ* is reported here. Three polymers show a monotonic electron mobility decrease as side‐chain size increasing due to the gradually lowered film crystallinity and change of backbone orientation. Interestingly, polymer PO12 with a moderate side‐chain size delivers a champion *σ* up to 92.0 S cm^−1^ and a power factor (PF) as high as 94.3 µW m^−1^ K^−2^ in the series when applied in OTE devices. The PF value is among the highest ones for the solution‐processing n‐doped polymers. In‐depth morphology studies unravel that the moderate crystallinity and the formation of 3D conduction channel derived from bimodal orientation synergistically contribute to high doping efficiency and large charge carrier mobility, thus resulting in high performance for the PO12‐based OTEs. The results demonstrate the great power of simple tuning of side chain in developing n‐type polymers with substantial *σ* for improving organic thermoelectric performance.

## Graphical abstract

A series of fused bithiophene imide dimer‐based polymers with distinct side‐chain size is developed to synergistically improve electron mobility and doping efficiency, which demonstrates remarkable electrical conductivity and power factor when applied in organic thermoelectrics.

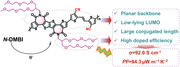

## 1 Introduction

Polymer semiconductors provide an excellent opportunity to function as low‐temperature (<200 °C) thermoelectric materials due to their advantages of solution processability, minimal toxicity, small thermal conductivity, and excellent mechanical flexibility,^[^ ^1^, ^2^, ^3^, ^4^, ^5^, ^6^ ^]^ enabling conformal coating at reduced device fabrication cost for thermoelectric generators as renewable thermal energy conversion devices, which are particularly important for mobile devices,^[^ ^7^ ^]^ wearable electronics,^[^ ^8^ ^]^ and sensor networks.^[^ ^9^, ^10^ ^]^ The thermoelectric performance is assessed by a dimensionless figure of merit of 𝑍𝑇 = 𝜎𝑆^2^𝑇/𝜅, where 𝜎, *S*, *T*, and *k* are the electrical conductivity, Seebeck coefficient, temperature, and thermal conductivity, respectively.^[^ ^6^, ^11^, ^12^ ^]^ To realize high‐performance organic thermoelectric modules, it is required for both p‐type and n‐type materials with high and comparable performance.^[^ ^13^ ^]^ To date, p‐type organic thermoelectric (OTE) materials, especially p‐type OTE polymers, have been intensively investigated and achieved the state‐of‐the‐art performance,^[^ ^14^, ^15^, ^16^, ^17^, ^18^, ^19^, ^20^ ^]^ exemplified by the benchmark p‐type polymers P3HT^[^ ^11^ ^]^ and poly(3,4‐ethylenedioxythiophene) with ZT > 0.4, which is originated from the high power factor (PF, PF = 𝜎𝑆^2^) of >300 µW m^−1^ K^−2^.^[^ ^21^, ^22^, ^23^, ^24^, ^25^, ^26^, ^27^ ^]^ Nevertheless, the n‐type counterparts lag greatly behind with much lower PFs typically ranging from 1 to 10 µW m^−1^ K^−2^, mainly suffering from the inferior electrical conductivities (𝜎s) typically in the range of 5–20 S cm^−1^. From the view of materials development, the unsatisfactory n‐type performance is mainly restrained by the scarcity of highly electron‐deficient building blocks with good solubility, compact molecular geometry, and optimized electronic properties.

At beginning, the widely used electron‐deficient building blocks were the historic imide‐functionalized arenes, i.e., naphthalene diimide (NDI)^[^ ^28^ ^]^ and perylene diimide (PDI).^[^ ^29^ ^]^ Their incorporation into donor–acceptor (D‐A) type polymers greatly improved the n‐type OTE performance at that time. However, the phenyl‐based NDI and PDI suffer from sizable steric hindrance with neighboring (hetero)arenes and hence twisted backbone in the resulting polymers, which significantly limits the electron mobility, thus leading to a poor 𝜎 (0.1–0.5 S cm^−1^) and PF (0.5–2 µW m^−1^ K^−2^).^[^ ^29^, ^30^, ^31^, ^32^ ^]^ Since then, a great number of electron‐deficient building blocks have been devised to improve the performance of OTEs of the resulting n‐type polymers (please see summary in Table S1, Supporting Information). They mainly include amide‐functionalized (hetero)arene‐based polymers,^[^ ^33^, ^34^, ^35^, ^36^ ^]^ imide‐functionalized (hetero)arene‐based polymers,^[^ ^37^, ^38^, ^39^ ^]^ boron‐nitrogen coordination bond‐based polymers,^[^ ^40^ ^]^ and ladder‐type polymers.^[^ ^30^, ^41^, ^42^, ^43^, ^44^ ^]^ For example, Pei et al. reported an amide‐functionalized n‐type polymer TBDOPV, which achieved a 𝜎 of 90 S cm^−1^ and a high PF of 106 µW m^−1^ K^−2^.^[^ ^44^ ^]^ Recently, Huang et al. reported a ladder‐type poly(benzodifurandione) (PBFDO) with low‐lying LUMO and good backbone coplanarity and found that it has ultrahigh conductivity of 2000 S cm^−1^ and a promising PF approaching 90 µW m^−1^ K^−2^.^[^ ^45^ ^]^ Nevertheless, the performance parameters of most polymers are still much inferior to their p‐type counterparts. Therefore, it is highly imperative to further design and synthesize high‐performance n‐type polymers for advancing the OTE field.

Based on the OTE performance parameters, it is clear that the low 𝜎 value is the major factor limiting the performance of n‐type organic and polymer semiconductors.^[^ ^46^, ^47^ ^]^ According to the equation: *σ* = *nµq*, where *n*, *µ*, and *q* are charge carrier concentration, charge carrier mobility, and elementary charge, respectively, hence both electron mobility and charge carrier concentration (i.e., n‐doping levels) are equally important to realize high *σ*s for n‐type polymers.^[^ ^48^ ^]^ Via backbone optimization, the resulting polymers could exhibit planar and linear backbone and high crystallinity, which results in improved charge transport in organic thin‐film transistors (OTFTs), but could be inclined to yield poor miscibility with dopant molecules and hence low doping level.^[^ ^49^, ^50^ ^]^ Therefore, it is highly challenging to simultaneously achieve high electron mobility and doping level to improve the *σ*s of n‐doped polymers.

**Figure 1:**
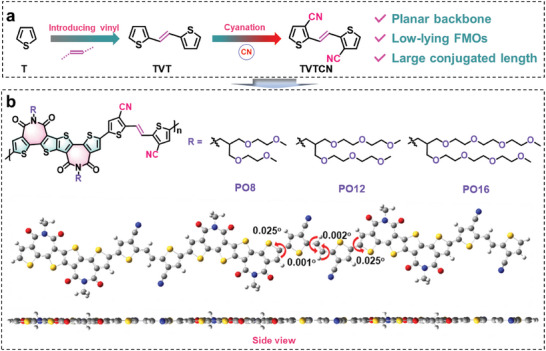
a) Molecular design strategy by increasing conjugation length and further incorporating cyano groups to yield TVTCN unit. b) Molecular structures of f‐BTI2g‐based polymers PO8, PO12, and PO16 decorated with distinct OEG‐type side‐chain 3‐((2‐methoxyethoxy)methyl)−2‐((2‐methoxyethoxy)methyl)propyl (O8), 3‐((2‐(2‐methoxyethoxy)ethoxy)methyl)−2‐((2‐(2‐methoxyethoxy)ethoxy)methyl)propyl (O12), and 13‐(2,5,8,11‐tetraoxadodecyl)−2,5,8,11‐tetraoxatetradecan‐14‐yl (O16) side‐chains (O16), respectively, and the optimized geometry of polymer backbone. Calculations were based on the DFT//B3LYP/6‐31G* level, and the alkyl side chains were substituted with the methyl group for calculation simplicity.

Bithiophene imide (BTI) and its derivatives^[^ ^51^ ^]^ have been proven to be the excellent electron‐deficient building blocks for accessing high‐performance n‐type polymers with large electron mobility (*µ*_e,OTFT_ > 3 cm^2^ V^−1^ s^−1^)^[^ ^52^ ^]^ achieved in OTFTs owing to their merits of excellent solubility, good backbone coplanarity, and high electron deficiency. Thus a great number of BTI derivatives have been invented to construct donor–acceptor (D‐A) and acceptor–acceptor (or all‐acceptor, A‐A) polymers for OTFTs^[^ ^53^, ^54^ ^]^ and all‐polymer solar cells.^[^ ^55^, ^56^ ^]^ For example, polymer f‐BTI2‐FT comprising of a fused bithiophene imide dimer (f‐BTI2) with alkyl side chains as acceptor unit and 3,4‐difluorothiophene as donor co‐unit showed unipolar n‐type character with a high *µ*_e,OTFT_ of 1.13 cm^2^ V^−1^ s^−1^ in OTFTs.^[^ ^54^ ^]^ When replacement of alkyl chain by oligo(ethylene glycol) (OEG)‐type chain, the minor structurally modified polymer f‐BTI2TEG‐FT maintained a high degree of backbone planarity and low‐lying lowest unoccupied molecular orbital (LUMO) energy level but with a much higher degree of n‐doping efficiency, thus resulting in an improved 𝜎 of ≈70 S cm^−1^.^[^ ^37^ ^]^ Nevertheless, the *µ*_e,OTFT_ is only 6.34 × 10^−4^ cm^2^ V^−1^ s^−1^, which limited the conductivity improvement.^[^ ^57^ ^]^ It is well known that the introduction of thienylene–vinylene–thienylene (TVT) unit can lead to increased conjugation length and more planar backbone compared to thiophene unit,^[^ ^58^ ^]^ demonstrating the advantages of improving microstructural ordering and charge transport in thin films for the resulting polymers (**Figure** **1**a). However, the electron‐rich nature of TVT limits polymer n‐type performance.^[^ ^59^ ^]^ On the other hand, cyano functionalization has been demonstrated as a powerful strategy to construct n‐type small molecules and polymers with low‐lying LUMOs, which improves the n‐type performance of various organic electronic devices, such as organic solar cells,^[^ ^60^ ^]^ OTFTs,^[^ ^61^ ^]^ and organic electrochemical transistors.^[^ ^62^ ^]^

Based on the great success of cyanation for polymers with improved electron transport properties and TVT with minimized steric hindrance, cyano‐functionalized TVT (TVTCN, Figure 1a) is expected to enable the resulting polymers with planar backbone, low‐lying LUMOs, and long conjugated length (Figure 1a).^[^ ^63^ ^]^ In addition to the polymeric backbone, the side‐chain also shows a significant effects on polymer chain stacking and film crystallinity, thus greatly influencing the device performance. In this contribution, we systematically study the impact of side‐chain size on the polymer optical/electrochemical properties, charge transport characters, n‐doping efficiency, and OTE performance. As shown in Figure 1b, PO8, PO12, and PO16 were attached with different branched OEG side chain, 3‐((2‐methoxyethoxy)methyl)−2‐((2‐methoxyethoxy)methyl)propyl (O8), 3‐((2‐(2‐methoxyethoxy)ethoxy)methyl)−2‐((2‐(2‐methoxyethoxy)ethoxy)methyl)propyl (O12), and 13‐(2,5,8,11‐tetraoxadodecyl)−2,5,8,11‐tetraoxatetradecan‐14‐yl (O16), respectively. The building blocks f‐BTI2g(O8), f‐BTI2g(O12), and f‐BTI2g(O16) were copolymerized with the same donor unit TVTCN to afford polymers PO8, PO12, and PO16 (Figure 1b), respectively. Intriguingly, three polymers show distinct optical/electrochemical properties and semicrystalline character. A monotonic mobility decrease with increasing side‐chain size was observed from PO8 to PO12 and to PO16 when applied in OTFTs. Among them, OTE devices based on polymer PO12 with moderate OEG side‐chain achieved a highest 𝜎 of 92 S cm^−1^ as well as a remarkable PF of 94.3 µW m^−1^ K^−1^, much higher than the devices based on polymers PO8 (𝜎 = 18.1 S cm^−1^ and PF = 14.7 µW m^−1^ K^−1^) and PO16 (𝜎 = 40.5 S cm^−1^ and PF = 62.1 µW m^−1^ K^−1^). To the best of our knowledge, the PF of 94.3 µW m^−1^ K^−1^ is among the highest values of n‐doped polymers for OTEs. The morphology analysis unravels that PO12 has a moderate crystallinity with bimodal orientation (both face‐on and edge‐on), which are beneficial to achieving high doping level and large electron mobility simultaneously, thus yielding high OTE performance. Overall, our work demonstrates that synergistical optimization of backbone orientation and side‐chain size provides a powerful avenue to develop n‐type polymers for high‐performance OTEs.

## 2 Results and Discussion

### 2.1 Chemical Synthesis

Figure S1 (Supporting Information) illustrates the synthetic routes to the key dibrominated monomers f‐BTI2g‐2Br(O8), f‐BTI2g‐2Br(O12), and f‐BTI2g‐2Br(O16), which are then reacted with distannylated monomer (*E*)−1,2‐bis(‐(3‐cyano‐5‐(trimethylstannyl)thiophene‐2‐yl)ethene (TVTCN‐2Sn) under typical Stille coupling‐based polycondensation to afford product polymers PO8, PO12, and PO16 with a yield of 74%, 81%, and 85%, respectively. The synthetic details can be found in the Supporting Information. After polymerization, the polymers were precipitated into methanol and then purified by successive Soxhlet extraction to afford the final polymer products. Using gel permeation chromatography (GPC) with hexafluoroisopropanol (HFIP) as the eluent, the number average molecular weight (*M*_n_)/dispersity (*Đ*) was found to be 11.7 kDa/1.6, 23.8 kDa/1.9, and 33.1 kDa/2.1 for PO8, PO12, and PO16, respectively (Figures S1–S3, Supporting Information). It was found that PO16 has the highest *M*_n_, attributed to the longest triethylene glycol side chains which offered the highest solubility among all polymers as the polymeric backbone extended during polymerization. Thermogravimetric analysis (TGA) unraveled that three polymers have superior thermal stability with a decomposition temperature over 270 °C, and differential scanning calorimetry (DSC) analysis showed no distinctive transition peaks from 30 to 250 °C for all three polymers (Figure S4, Supporting Information).

### 2.2 Polymer Optical and Electrochemical Properties

**Figure 2:**
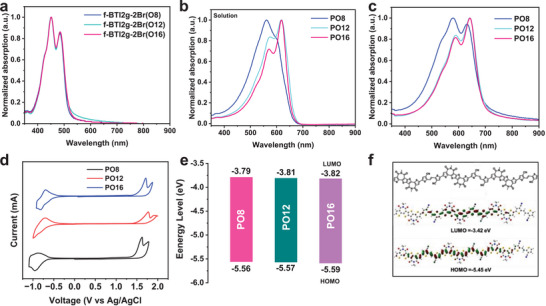
Normalized UV–vis absorption spectra of a) the dibrominated monomer films, and f‐BTI2‐based polymers b) in diluted chloroform solution (10^−5^ m) and c) as thin films. d) Cyclic voltammograms of the polymer thin films in 0.1 m tetra(*n*‐butyl)ammonium hexafluorophosphate acetonitrile solution with Fc/Fc^+^ as the external standard. e) FMO energy‐level alignment of polymers. f) Optimized molecular geometries for trimer of the polymer repeating unit. The calculations were performed at the B3LYP/6‐31G(d,p) level, and the alkyl chains were replaced with a methyl group for calculation simplicity.

The absorption properties of f‐BTI2g‐2Br(O8), f‐BTI2g‐2Br(O12), and f‐BTI2g‐2Br(O16) in chloroform solution and as thin film are depicted in Figure S5 (Supporting Information) and **Figure** **2**a, and the related absorption parameters are summarized in **Table** **1**. Three monomers in solution show comparable absorption spectra with the maximum absorption wavelengths (*λ*_max_) located at 463 nm. From solution to film state, all monomers exhibit a redshift of ≈10 nm as a result of the increased backbone planarity and chain aggregation. After polymerized with TVTCN unit, three polymers show distinct absorption features in the range of 400–700 nm. For PO8, a strong absorption peak is found at 561 nm in solution, associated with the intramolecular charge transfer (ICT) between f‐BTI2 and TVTCN units, while both PO12 and PO16 featured similar bathochromically shifted absorption peaks at 571 nm. Compared to solution, the *λ*_max_ of three polymer films exhibited a pronounced redshift of ≈20 nm due to the fact that they have a higher degree of polymer chain ordering in film state. The polymer optical bandgaps (*E*_g_^opt^) were determined from the absorption onset of polymer films, corresponding to 1.79, 1.77, and 1.77 eV for PO8, PO12, and PO16, respectively.

**Table 1:** Optical and electrochemical properties of the f‐BTI2‐based polymer semiconductors

Polymer	*M*_n_ [kDa]	*Đ*	*λ*_max_^soln^ ^a)^ [nm]	*λ*_max_^film^ ^b)^ [nm]	*λ*_onset_^film^ [nm]	*E*_g_^opt^ ^c)^ [eV]	*E*_LUMO_ ^d)^ [eV]	*E*_HOMO_ ^e)^ [eV]
PO8	11.7	1.6	560	579	693	1.79	−3.78	−5.56
PO12	23.8	1.9	617	641	699	1.77	−3.81	−5.57
PO16	33.1	2.1	617	641	699	1.77	−3.82	−5.59

^a)^ Absorption of polymer solution (10^−5^ M in chloroform)

^b)^ Absorption of as‐cast polymer film from chloroform solution

^c)^ Optical bandgap (*E*_g_^opt^) derived from absorption onset of polymer film using the equation: *E*_g_^opt^ = 1240/*λ*_onset_^film^ (eV)

^d)^ *E*_LUMO_ = −(*E*_red_^onset^ + 4.80) eV, *E*_red_^onset^ determined using the Fc/Fc^+^ external standard

^e)^ *E*_HOMO_ = *E*_LUMO_ − *E*_g_^opt^.

Please note that PO12 and PO16 demonstrate significantly bathochromically shifted absorption compared to PO8 in both solution and film states. The temperature‐dependent UV–vis absorption was characterized to investigate the polymer aggregation properties. As shown in Figure S6 (Supporting Information), the peak of PO8 gradually disappeared when the temperature was increased to 90 °C, whereas PO12 and PO16 exhibited stronger pre‐aggregation character (Figures S7–S8, Supporting Information) compared to PO8 with smaller side‐chain size even in dilute chlorobenzene, suggesting the stronger intermolecular interaction of the longer OEG side chains (O12 and O16 side chains) compared with O8 side chain. The strong aggregation tendency of polymers PO12 and O16 corroborates the observed redshifted absorption spectra and narrower *E*_g_^opt^.

The frontier molecular orbital (FMO) energy levels of three polymers were probed by cyclic voltammetry (CV) with Ag/Ag^+^ as the reference electrode and ferrocene/ferrocenium (Fc/Fc^+^) redox couple as the external standard in 0.1 m tetra(*n*‐butyl)ammonium hexafluorophosphate acetonitrile solution (Figure 2d), and Table 1 summarizes the corresponding parameters. The LUMO/highest occupied molecular orbital (HOMO) energy levels were found to be −3.79/−5.56, −3.81/−5.57, and −3.82/−5.59 eV for PO8, PO12, and PO16, indicative of a negligible effect of lateral side‐chain size on their electrochemical properties. In order to understand polymer backbone topology and FMOs, the density functional theory (DFT) calculation was conducted using trimer of the polymer repeating unit, and the long side chains were replaced by shorter methyl groups. As shown in Figure 2f, the polymer exhibits a minimum dihedral angle of ≈1° between BTI2 unit and the neighboring thiophene moieties, indicating a very planar backbone of the f‐BTI2‐based polymer.

### 2.3 Polymer Charge Transport Property

**Figure 3:**
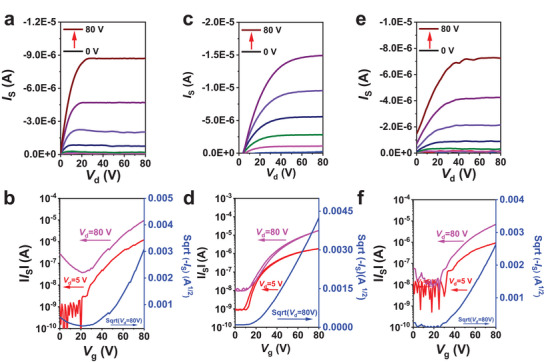
OTFT output (top) and transfer (bottom) characteristics of the f‐BTI2g‐based polymers: a,b) PO8, c,d) PO12, and e,f) PO16.

The charge transport properties of three polymers are studied by fabricating top‐gate/bottom‐contact (TG/BC) OTFTs with a device structure of glass/Au/polymer/CYTOP/Al, in which a fluorinated perfluoro(1‐butenyl vinyl ether) polymer (CYTOP) is used as dielectric layer. The details of device fabrication are depicted in the Supporting Information, and the output/transfer characteristics of the optimal devices are displayed in **Figure** **3**. All polymers exhibit nearly unipolar n‐type transport character without using any electrode‐modifying layer, originated from their deep‐lying LUMO/HOMO levels. It was found that the electron mobilities decrease monotonically with the increase of side‐chain size. After the systematical optimizations, PO8 delivered a remarkable *µ*_e,OTFT_ of 0.013 ± 0.02 cm^2^ V^−1^ s^−1^, which was slightly higher than both PO12 (*µ*_e,OTFT_ = 0.010 ± 0.03 cm^2^ V^−1^ s^−1^) and PO16 (*µ*_e,OTFT_ = 0.007 ± 0.001 cm^2^ V^−1^ s^−1^), indicative of a large influence of OEG chain size on charge transport in OTFTs.^[^ ^64^ ^]^ The OTFT mobilities of ≈0.01 cm^2^ V^−1^ s^−1^ achieved from PO8 device are among the highest values for n‐type polymers functionalized with OEG‐type side chains, which could be beneficial to obtaining high electron mobility after doping. Please note that these OTFT mobilities are significantly less than those analogues with alkyl side chains.^[^ ^54^ ^]^ We guess that the OEG side chain would induce water‐related electron traps because of its hydrophilicity and result in widening interfacial charge transport density of states due to its high polarity.^[^ ^65^ ^]^

### 2.4 n‐Doping of the Polymers

**Figure 4:**
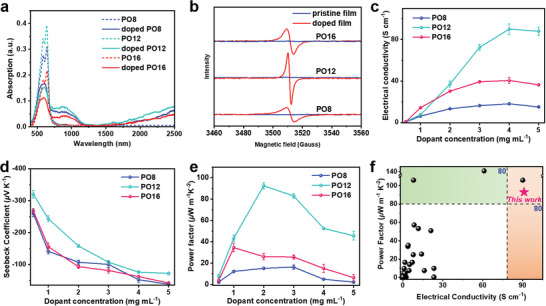
a) UV–vis–NIR and b) electron paramagnetic resonance (EPR) spectra of the pristine and *N*‐DMBI‐doped polymer films. c) Electrical conductivity, d) Seeback coefficient, and e) power factor of three polymers‐based OTE devices as function of dopant concentration. f) Electron conductivity and power factor values of the f‐BTI2g‐based polymers together with high‐performance n‐type polymers reported in literature.

To explore n‐doping properties, UV–vis–NIR absorption spectra of three polymers were first investigated. The polymer films were doped by sequential protocol instead of traditional blend doping due to its advantage of achieving more ordered film morphology of doped polymer.^[^ ^66^, ^67^, ^68^, ^69^ ^]^ *N*,*N*‐Dimethyl‐2‐phenyl‐2,3‐dihydro‐1H‐benzoimidazole (*N*‐DMBI) was selected as the dopant for three polymers due to its good air‐stability, strong reducing ability, and excellent solution processability.^[^ ^70^, ^71^ ^]^ As shown in **Figure** **4**a, three doped polymer films exhibited two typical (bi)polaron absorption bands in the low energy band region (800–2500 nm) accompanied by a weak ICT absorption peak in the range of 400–700 nm, indicating that *N*‐DMBI has successfully doped three polymers. Compared to PO8 and PO16, PO12 exhibits much stronger (bi)polaron absorption, which suggests more efficient n‐doping of PO12 film.

Electron paramagnetic resonance (EPR) spectroscopy (Figure 4b) was further carried out to estimate the number of radicals formed in the doped films, and the doped PO12 film showed a stronger EPR intensity compared to PO8 and PO16, which likely indicates a higher doping efficiency of PO12. The results of UV–vis–NIR spectra and EPR spectroscopy unraveled a higher doping level of PO12, which was expected to yield higher 𝜎 (vide infra).

### 2.5 Polymer Thermoelectric Performance

Thermoelectric devices were fabricated and characterized in glovebox to minimize interference by O_2_/water de‐doping. The optimal device performance of PO8 and PO16 was achieved using chloroform as the solvent, and the optimal performance PO12 was attained using hexafluoroisopropanol. The *N*‐DMBI concentration was systematically varied from 0.1 0.5, 1, 2, 3, 4 to 5 mg mL^−1^, and the related performance parameters are summarized in Figure 4c–e and **Table** **2**. Three pristine polymers showed very low intrinsic conductivity in accordance with their low‐lying HOMO/LUMO levels.^[^ ^49^ ^]^ After doping with a low dopant concentration of 0.1 mg mL^−1^, the conductivity increases substantially due to the generation of mobile charge carriers. With a further increase of dopant concentration, the conductivity was gradually enhanced, and it was found that all three polymers yield a saturated conductivity at 4 mg mL^−1^ *N*‐DMBI concentration. The average conductivities are found to be 17.5, 90.1, and 40.1 S cm^−1^ for PO8, PO12, and PO16, respectively. The highest 𝜎 of PO12 is over five and two times higher than those of both PO8 and PO16, respectively. In order to highlight the crucial role of sequential doping, we also performed blending doping for three polymers. As shown in Figure S12 (Supporting Information), doped PO8, PO12, and PO16 films achieved the highest 𝜎s of 14.5, 51.6, and 23.5 S cm^−1^, respectively, at optimal dopant ratio. Hence, compared to sequential doping, the blend doping leads to the inferior conductivity, mainly attributed to the reduced ordering of polymer morphology when blended with dopant.

**Table 2:** Electron mobilities and n‐type thermoelectric performance parameters of polymers PO8, PO12, and PO16‐based OTE devices

Polymer	*µ*_e,OTFT_ ^a)^ [cm^2^ V^−1^ s^−1^]	*σ* ^b)^ [S cm^−1^]	*S* ^c)^ [µV K^−1^]	PF^c)^ [µW m^−1^ K^−2^]
PO8	0.015 (0.013 ± 0.02)	18.1 (17.5 ± 0.4)	−110.2 (−107.2 ± 2)	14.7 (13.1 ± 1.2)
PO12	0.013 (0.010 ± 0.03)	92.0 (90.1 ± 3)	−162.6 (−158 ± 2)	94.3 (92.5 ± 2)
PO16	0.007 (0.005 ± 0.001)	43.5 (40.1 ± 0.5)	−95.4 (−92.1 ± 1)	62.1 (57.8 ± 4)

^a)^ Electron mobility from OTFT devices

^b)^ The conductivity of the polymers n‐doped at the optimal *N*‐DMBI concentration

^c)^ The power factors and the corresponding Seebeck coefficient values. The average values in parenthesis are from at least five devices.

To gain insight into the *σ* difference for three polymers, the carrier concentration (*n*) and mobility (*µ*_e,doping_) were obtained by AC magnetic field Hall measurements. At optimized doping concentration, the doped PO12 exhibited a significantly higher carrier concentration of 9.04 ± 0.4 × 10^20^ cm^−3^ in comparison to two other polymers (PO8: 6.18 ± 0.1 × 10^20^ and PO16: 8.30 ± 0.1 × 10^20^) (Table S3, Supporting Information). On the other hand, the *µ*_e,doping_ of doped PO12 film reached 0.64 ± 0.3 cm^2^ V^−1^ s^−1^, which is also larger than those of PO8 (0.18 ± 0.2 cm^2^ V^−1^ s^−1^) and PO16 (0.33 ± 0.2 cm^2^ V^−1^ s^−1^). The results demonstrate that the doped PO12 film achieved efficient charge transport and large mobile carrier concentration synchronously, which directly contributed to the higher conductivity. Furthermore, the doping efficiencies of three polymers at optimized doping concentration were estimated to be 37.6%, 63.3%, and 66.4% for PO8, PO12, and PO16 films, respectively, based on the carrier concentration. Note that the values of doping efficiencies may not be the real ones due to the great challenge of obtaining the densities for three polymers, the comparison of the relative trends should be meaningful. For PO12 and PO16, the large values of doping efficiencies indicate higher doping levels, which is likely originated from both relatively low crystallinity benefiting the penetration of dopant molecules (vide infra).

The Seebeck coefficient (*S*) of the doped polymer films was further probed by systematically varying temperature gradient (Δ*T*) across the samples from 0 to 2.8 K with a step of 0.7 K then back to 0 K. The related *S* and calculated PF values are summarized in Figure 4d and Table 2. The *S* values gradually decrease as *N*‐DMBI concentration increases due to the fact that *S* is in negative correlation with charge carrier concentration.^[^ ^30^, ^33^ ^]^ As a result, low dopant concentration delivered significantly higher *S* as compared to high concentration. By balancing *σ* and *S*, the highest PFs are achieved at 1, 2, and 1 mg mL^−1^ concentration of *N*‐DMBI for PO8, PO12, and PO16, affording the value of 13.1 ± 1.2, 92.5 ± 2, and 57.8 ± 4 µW m^−1^ K^−2^, respectively. Clearly, the PO12‐based devices attained the highest OTE performance than the shorter branched side‐chain functionalized PO8‐ and longer side‐chain functionalized O16‐based devices, attributed to the optimized morphology (vide infra), large charge mobility, and high doping efficiency of polymer PO12. To the best of our knowledge, the PF of 94.3 µW m^−1^ K^−2^ and conductivity of 92 S cm^−1^ are among the highest values for the solution‐processed n‐doped polymers (Figure 4f and Table S1, Supporting Information), and the results manifest that the side‐chain engineering is a powerful strategy to develop high‐performance OTE polymers. The stability of n‐type thermoelectric devices is a crucial factor for practical application, which was investigated by detecting the variation of electrical conductivity of these doped films in the air (Figure S13, Supporting Information). For these polymers, it was found that their electrical conductivities rapidly dropped by four orders of magnitude to be around 10^−4^ S cm^−1^ after 4 h storage in the air. The relatively high‐lying LUMOs are the main reason of the fast decay behavior, highlighting the importance of low‐lying LUMO for enabling stable thermoelectric devices.

### 2.6 Film Morphology and Molecular Packing

**Figure 5:**
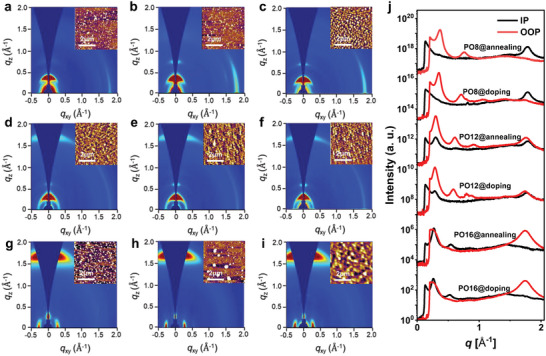
2D GIWAXS images and AFM height images (inset) of a–c) PO8, d–f) PO12, and g–i) PO16 films. The as‐cast, annealed, and n‐doped films are corresponding to (a, d, g), (b, e, h), and (c, f, i), respectively. j) The corresponding in‐plane and out‐of‐plane line‐cut profiles of the 2D GIWAXS images of the polymers.

To gain insights into the correlation between the polymer chemical structure and OTE performance, the morphology of the pristine, annealed, and doped thin films were investigated by atomic force microscopy (AFM) first. As displayed in **Figure** **5**, three pristine films show relatively rough surfaces with smooth root‐mean‐square (RMS) roughness values of ≈2 nm, whereas the annealed ones feature smoother and more uniform surface with RMS roughness values of ≈1 nm. The RMS roughness of doped films became larger with the values of 20.5, 8.6, and 3.1 nm for PO8‐, PO12‐, and PO16‐based doped films, respectively, likely originated from the sequential doping strategy adopted. The dopants might incline to aggregate on the film surfaces. Interestingly, the n‐doped PO12 and PO16 films exhibited smaller RMS roughness than that of PO8, demonstrating that the better miscibility of both polymers with the dopant molecule is ascribed to the large OEG side‐chain size of PO12 and PO16, which should be contributed to the higher doping efficiency.

Two‐dimensional grazing incidence wide‐angle X‐ray scattering (2D‐GIWAXS) characterization was further employed to investigate film crystallinity and polymer packing. As shown in Figure 5a, the pristine PO8 film adopts an edge‐on orientation with a strong hump‐shaped (100) diffraction peak having a *q*_z_ value of 0.37 Å^−1^ (corresponding to a lamellar stacking distance of 17.0 Å) in out‐of‐plane (OOP) direction and a pronounced (010) diffraction peak at 1.79 Å^−1^ (corresponding to a π–π stacking distance of 3.51 Å) in plane (IP) direction.

After thermal annealing, higher order lamellar diffraction (200) emerged in the OOP direction and a stronger (010) π–π stacking diffraction peak in the IP direction can be found for annealed PO8, indicative of the improved film crystallinity in comparison to as‐cast film. For annealed PO12 film, it was found that the lamellar diffractions also progress up to (200) in the OOP direction and obvious (010) diffraction peaks appear in both OOP and IP directions, suggesting bimodal orientation with both face‐on and edge‐on fractions. Unexpectedly, the orientation of annealed PO16 changes to face‐on orientation with a broadened (010) diffraction peak along the OOP direction at *q*_z_ = 1.74 Å in combination with a (100) scattering at *q*_xy_ = 0.27 Å^−1^ (lamellar stacking distance of 23.3 Å) in IP direction. Among them, PO8 exhibited an edge‐on orientation with the strongest crystallinity, which matches well with its highest electron mobility in OTFTs.^[^ ^72^ ^]^ After doping, three films still maintained the orientations of the annealed ones, indicating negligibly disrupted crystalline domain and molecular orientation using sequential doping strategy. The existence of multiple parallel and perpendicular orientations of polymer chains which can form 3D conduction channels is conducive to high charge mobility in OTE devices due to the fact that grain boundaries (or traps and defects) exist in polymer microstructures.^[^ ^18^, ^73^ ^]^ The relatively large ravine of grain boundaries (or traps and defects) exist in polymer PO12 microstructures compared with PO8 and PO16 with single orientations would be beneficial for dopant permeation, thus leading to the fast doping process. Moreover, PO12 and PO16 with the relatively low crystallinity and large packing distance of polymer chains (both lamellar and π–π stacking distances) should better accommodate dopant molecules than PO8 after doping due to the fact that the dopants or dopant cations tend to exist and migrate in the amorphous regions and irregular domains.^[^ ^18^, ^73^ ^]^ Thus the 3D conduction channels together with appropriate crystalline phase of polymer PO12 can synchronously deliver fast charge transport and high n‐doping efficiency, thus leading to the large electrical conductivity and PF.

**Figure 6:**
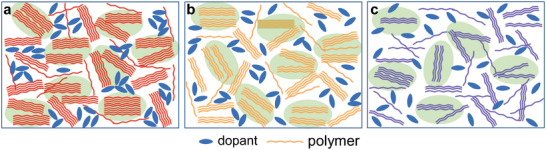
Schematic of molecular packing of a) PO8, b) PO12, and c) PO16 films with dopant.

To better illustrate the topographic differences of doping process between three polymers and dopant, **Figure** **6** shows the schematics of the film morphology after doping. As compared to PO12 and PO16, PO8 exhibits larger domains due to the stronger crystallization tendency. During n‐doping by the sequential protocol, dopant molecules may not readily penetrate into large and highly crystalline domains for PO8 film, thus inclining to form pure dopant domains. In contrast, PO16 with the largest OEG side‐chain size presents the lowest crystallinity and forms the smallest domains, which can facilitate the diffuse of dopant molecules, thus resulting in the highest polymer:dopant miscibility. However, this could be detrimental to forming effective charge transport channels between small domains. For PO12 with medium side‐chain size, it has relatively moderate crystallinity and stacking distance compared with PO8 and PO16, making that it not only has crystalline but not so over‐size domains. After doping, dopants can successfully infiltrate into PO12 domains and form highly doped polymer film with well interconnected matrix. Hence, PO12 can keep efficient charge transport network with 3D conduction channels, which is beneficial to yielding high electrical conductivity after doping. As a result, the n‐doped PO12 can realize high charge carrier mobility and high doping efficiency, simultaneously, by optimizing the film crystallinity and polymer chain orientation, thus resulting in excellent OTE performance.

## 3 Conclusion

In conclusion, we designed and synthesized three fused bithiophene imide dimer‐based n‐type polymers PO8, PO12, and PO16 decorated with distinct OEG side‐chain. Varying the length of side chains enables us to tune the film crystallinity and packing orientation of polymer chains. It was found that PO12 and PO16 with larger side‐chains show relatively redshifted absorption compared with PO8 and the FMOs slightly become lower‐lying with the increase of side‐chain size. When applied in OTFTs, PO8, PO12, and PO16 exhibited a monotonic mobility decrease from 0.015 to 0.013 and to 0.008 cm^−2^ V^−1^s^−1^ with gradual increase of side‐chain size. For polymer PO8, the achieved highest mobility is attributed to its close molecular packing and favorable edge‐on orientation as evidenced by 2D‐GIWAXS characterizations. Interestingly, PO12 with the medium side‐chains has moderate crystallinity and bimodal orientation, benefiting to penetration of the dopant into polymer, which leads to formation of well interconnected matrix with efficient charge transport network and 3D conduction channels. Therefore, PO12 exhibited a highest n‐doping efficiency when n‐doped with *N*‐DMBI. As a consequence, PO12 delivered excellent electrical conductivity of 92.0 S cm^−1^ and power factor of up to 94.3 µW m^−1^ K^−1^, both of which are among the highest value for n‐type polymers. Overall, our systematic study demonstrates that tuning the oligo(ethylene glycol)‐type side‐chain size is a very straightforward and powerful approach to equilibrating the doping level and charge mobility, leading to improved conductivity and thus promoting the OTE performance. We believe that these findings provide important guidelines to develop efficient n‐type polymers to advance of the OTE field.

## Conflict of Interest

The authors declare no conflict of interest.

## Supporting information

Supporting Information

## Data Availability

The data that support the findings of this study are available from the corresponding author upon reasonable request.
