# Supplementary material for: High‐Performance n‐Type Organic Thermoelectrics Enabled by Synergistically Achieving High Electron Mobility and Doping Efficiency
Source: Adv Sci (Weinh). 2023 Aug 8;10(29):2302629. doi: 10.1002/advs.202302629 (PMC10582446; doi:10.1002/advs.202302629)
Supplement: Supplementary file 1 — Supporting Information [file ADVS-10-2302629-s001.PDF]

Supporting Information

High-Performance n-Type Organic Thermoelectrics Enabled by Synergistically Achieving High Electron Mobility and Doping Efficiency

*Kui Feng, Junwei Wang, Sang Young Jeong, Wanli Yang, Jianfeng Li, Han Young Woo, Xugang Guo**

Dr. K. Feng, J. Wang, W. Yang, J. Li, Prof. X. Guo
Department of Materials Science and Engineering, Southern University of Science and Technology, Shenzhen, Guangdong 518055, China
E-mail: guoxg@sustech.edu.cn

Dr. K. Feng
Academy for Advanced Interdisciplinary Studies, Southern University of Science and Technology, Shenzhen, Guangdong 518055, China
S. Y. Jeong, Prof. Y. H. Woo

Department of Chemistry, Korea University, Anamro 145, Seoul 02841, Republic of Korea

Table of Contents

1. Materials Synthesis

2. Materials Characterization

3. Supplementary Tables

4. Supplementary Figures

1. Materials Synthesis

The synthetic routes to the monomers f-BTIg-2Br(O8), f-BTIg-2Br(O12) and f-BTIg-2Br(O16), and their resultant polymers are illustrated in Scheme S1. The reagents and chemicals were purchased from Admas-beta, and Bide pharmatch Ltd., *etc*, and the monomer TVTCN-2Sn were synthesized according to the literature.[1] The polymers were purified by Soxhlet extraction and characterized by ^{1}H NMR and elemental analysis.

Scheme S1. Synthesis routes to the monomers f-BTI2g-2Br(O8), f-BTI2g-2Br(O12), and f-BTI2g-2Br(O16).

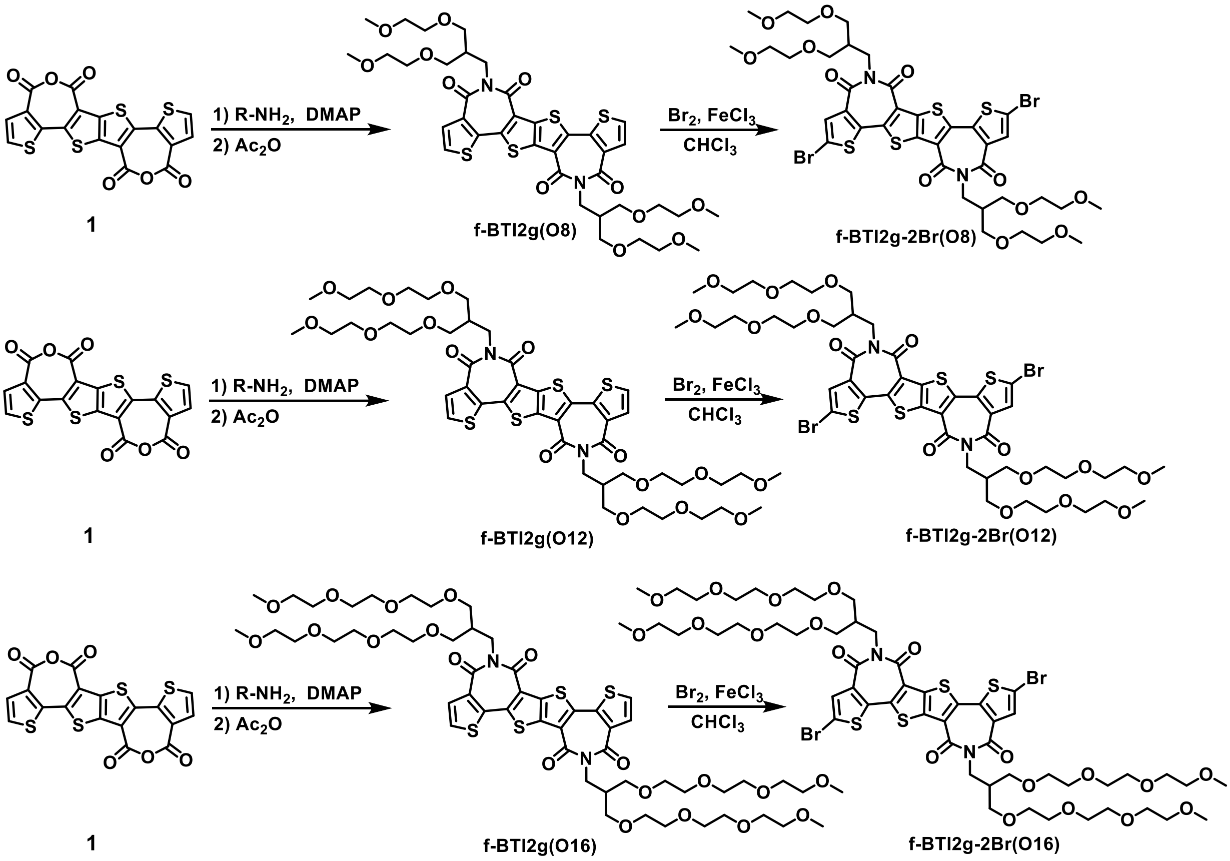

Scheme S2. Synthesis routes to the polymers PO8, PO12, and PO16.

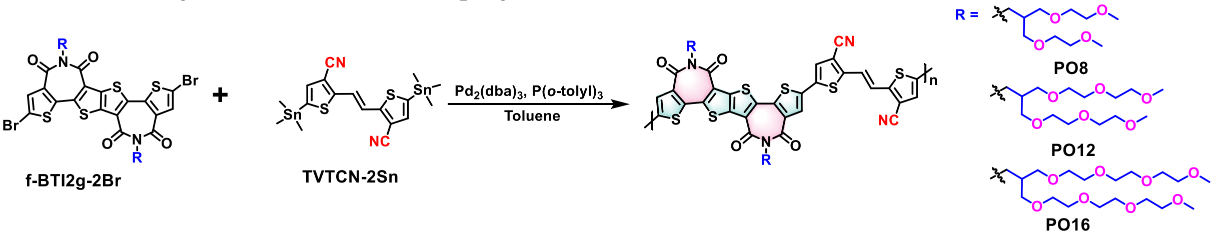

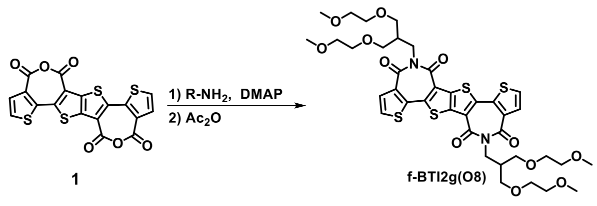

Synthesis of compound ***f-BTI2g(O8)***

4-Dimethylaminopyridine (DMAP, 0.33 g, 2.73 mmol) and R-NH_2 (0.45 g, 2.05 mmol) were added to a suspension of compound **1** (0.30 g, 0.68 mmol) in 20 mL anhydrous 1,4-dioxane. The reaction mixture was stirred and heated to 80 °C overnight. After cooled to room temperature, 5 mL acetic anhydride was added via syringe in one portion. The reaction was further heated to 140 °C for 3 h. After cooled to room temperature, the reaction mixture was extracted with CH_2Cl_2 three times and the combined organic layer was dried over Na_2SO_4. The solvent was removed under a reduced pressure to afford a residue, which was further purified by column chromatography over silica gel using dichloromethane:methanol (80:1) as the eluent to give the f-BTI2g(O8) (0.46 g, yield: 80%). ^{1}H NMR (400 MHz, $CDCl_3$) δ (ppm): 7.80-7.79 (d, J = 13.4 Hz, 2H), 7.33-7.31 (d, J = 13.4 Hz, 2H), 4.47-4.45 (d, J = 17.5 Hz, 4H), 3.63-3.51 (m, 16H), 3.46-3.36 (m, 8H), 3.29 (s, 12H), 2.61-2.54 (m, 2H). ^{13}C NMR (100 MHz, $CDCl_3$) δ(ppm): 161.20, 161.13, 141.33, 140.00, 137.89, 134.05, 133.35, 125.33, 124.15, 71.80, 71.13, 70.44, 59.01, 45.84, 38.02.

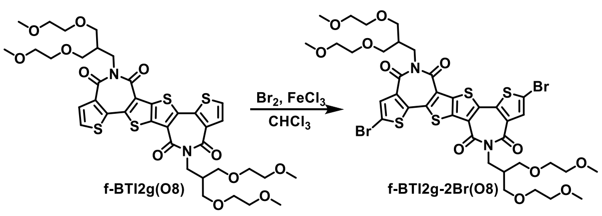

Synthesis of monomer ***f-BTI2g-2Br(O8)***

Br_2 (0.13 g, 0.78 mmol) in 2 mL chloroform was added dropwise into a solution of compound f-BTI2g(O8) (0.26 g, 0.31 mmol) and $FeCl_3$ (0.005 g) in 10 mL chloroform at 0 °C. The reaction was warmed to room temperature and stirred overnight. 50 mL water was then

added, and organic layer was extracted with CH_2Cl_2 for 3 times. The combined organic solvent was removed under reduced pressure. The residue was further purified by silica column chromatography using dichloromethane:methanol (100:1) as the eluent to give f-BTI2g-2Br(O8) as a yellow solid (0.26 g, yield: 83%). Anal. calcd for $C_{38}H_{44}Br_2N_2O_{12}S_4$ (%): C, 45.24; H, 4.40; N, 2.78; S, 12.71. Found (%): C, 45.31; H, 4.21; N, 2.49; S, 12.50. ^{1}H NMR (400 MHz, $CDCl_3$) δ (ppm): 7.73 (s, 2H), 4.44-4.42 (d, J = 17.3 Hz, 4H), 3.60-3.37 (m, 24H), 3.28 (s, 12H), 2.56-2.50 (m, 2H). ^{13}C NMR (100 MHz, CDCl3) δ (ppm): 160.66, 159.98, 140.00, 139.02, 138.05, 136.21, 133.58, 124.17, 113.57, 71.80, 71.13, 70. 44, 59.03, 46.01, 37.92.

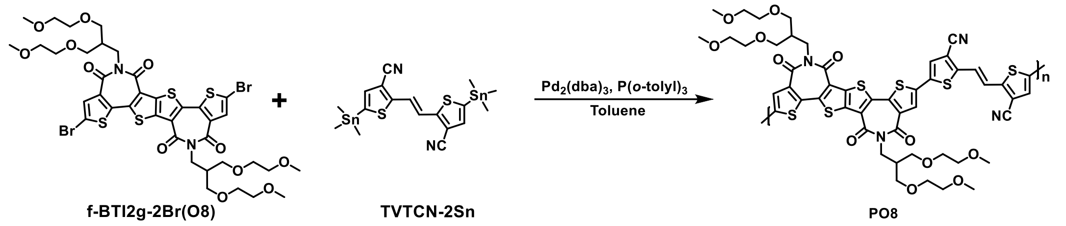

Synthesis of polymer PO8

To a 5 mL vial was charged with monomer f-BTI2g-2Br(O8) (0.10 g, 0.1 mmol), 2,5-bis(trimethylstannyl)thiophene (TVTCN-2Sn) (0.057 g, 0.1 mmol), tris(dibenzylideneacetone) dipalladium(0) ($Pd_2(dba)_3$, 4.0 mg), and tris(*o*-tolyl)phosphine (P(*o*-tolyl)$_3$, 10 mg). The vial was subjected to 3 pump/purge cycles with argon before anhydrous toluene (3 mL) was added. The vial was sealed under argon flow and then heated at 140 °C by microwave irradiation for 3 h. After cooled down to room temperature, the reaction mixture was dripped into 100 mL methanol. The precipitates were filtrated and further purified by Soxhlet extraction with methanol, acetone, hexane, dichloromethane, and chloroform, sequentially. The final chloroform fraction was concentrated and dripped into methanol. The precipitate was collected and dried to afford product polymer PO8 (0.081g, yield: 74%) as a black solid. Anal. calcd. for $C_{50}H_{48}N_4O_{12}S_6$ (%): C, 55.13; H, 4.44; N, 5.14; S, 17.66. Found (%): C, 55.23; H, 4.32; N, 5.32; S, 17.12. ^{1}H NMR (400 MHz, $CDCl_3$) δ (ppm): 7.80-7.77 (br, 2H), 7.11–7.09 (br, 2H), 4.14–4.11 (br, 4H), 3.53–3.49 (br, 24H), 3.20–3.16 (br, 12H), 2.46–2.41 (br, 2H).

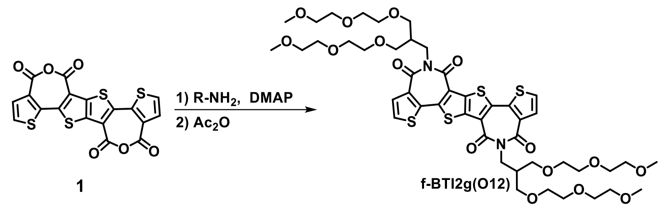

Synthesis of compound ***PO8***

4-dimethylaminopyridine (DMAP, 0.33g, 2.73 mmol) and R-NH_2 (0.63 g, 2.05 mmol) were added to a suspension of compound **1** (0.30 g, 0.68 mmol) in 20 mL anhydrous 1,4-dioxane. The reaction mixture was stirred and heated to 80 °C overnight. After cooled to room temperature, 5 mL acetic anhydride was added via syringe in one portion. The reaction was further heated to 140 °C for 3 h. After cooled to room temperature, the reaction mixture was extracted with CH_2Cl_2 three times and the combined organic layer was dried over Na_2SO_4. The solvent was removed under a reduced pressure to afford a residue, which was purified by column chromatography over silica gel using dichloromethane:methanol (100:1) as the eluent to give the f-BTI2g(O12) (0.25 g, yield: 80%). ^{1}H NMR (400 MHz, $CDCl_3$) δ (ppm): 7.83-7.78 (d, J = 5.3 Hz, 2H), 7.35-7.34 (d, 2H), 4.45-4.44 (d, 4H), 3.58-3.49 (m, 40H), 3.35 (s, 12H), 2.53-2.52 (m, 2H). ^{13}C NMR (100 MHz, $CDCl_3$) δ(ppm): 161.12, 161.05, 141.31, 137.88, 134.04, 133.32, 125.42, 124.09, 71.91, 586 71.00, 70.59, 70.52, 70.44, 59.02, 45.77, 38.20.

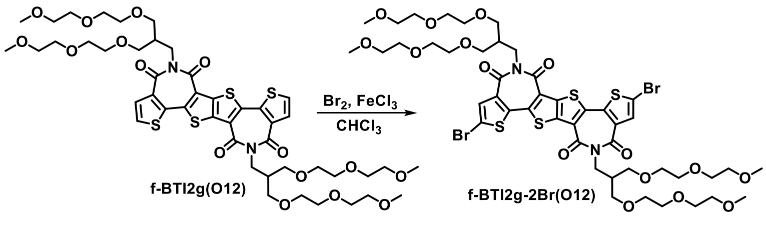

Synthesis of monomer ***f-BTI2g-2Br(O12)***

Br_2 (0.13 g, 0.78 mmol) in 2 mL chloroform was added dropwise into a solution of compound f-BTI2g(O12) (0.32 g, 0.31 mmol) and $FeCl_3$ (5 mg) in 5 mL chloroform at 0 °C. The reaction mixture was warmed to room temperature and stirred overnight. 50 mL water was then added and the mixture was extracted with chloroform for 3 times. The combined organic solvent was removed under reduced pressure. The residue was further purified by silica column

chromatography using dichloromethane:methanol (50:1) as the eluent. The product monomer f-BTI2g-2Br(O12) was obtained as a yellow solid (0.30 g, yield 83%). Anal. calcd for $C_{46}H_{60}Br_2N_2O_{16}S_4$ (%): C, 46.62; H, 5.10; N, 2.36; S, 10.82. Found (%): C, 45.64; H, 5.08; N, 2.35; S, 10.20. 1H NMR (400 MHz, CDCl3) δ (ppm): 7.81 (s, 2H), 4.47-4.46 (d, 4H), 3.61-3.50 (m, 40H), 3.37 (s, 12H), 2.57-2.50 (m, 2H). 13C NMR (100 MHz, CDCl3) δ (ppm): 160.81, 160.10, 140.14, 139.13, 138.15, 136.21, 133.62, 124.31, 113.65, 71.93, 71.01, 70.59, 70.55, 70.45, 59.06, 45.98, 38.14.

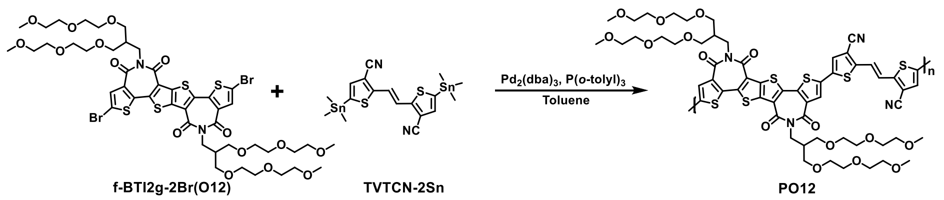

*Synthesis of polymer **PO12***

To a 5 mL vial was charged with monomer f-BTI2g-2Br(O12) (0.12 g, 0.1 mmol), 2,5-bis(trimethylstannyl)thiophene (TVTCN-2Sn) (0.057 g, 0.1 mmol), tris(dibenzylideneacetone) dipalladium(0) ($Pd_2(dba)_3$, 4.0 mg), and tris(*o*-tolyl)phosphine (P(*o*-tolyl)$_3$, 10 mg). The vial was subjected to 3 pump/purge cycles with argon before anhydrous toluene (3 mL) was added. The vial was sealed under argon flow and then heated at 140 ºC by microwave irradiation for 3 h. After cooled down to room temperature, the reaction mixture was dripped into 100 mL methanol. The precipitates were filtrated and further purified by Soxhlet extraction with methanol, acetone, hexane, dichloromethane, and chloroform, sequentially. The final chloroform fraction was concentrated and dripped into methanol. The precipitate was collected and dried to afford product polymer PO12 (0.11 g, yield: 81%) as a black solid. Anal. calcd. for $C_{56}H_{64}N_4O_{16}S_6$ (%): C, 55.04; H, 5.10; N, 4.43; S, 15.20. Found (%): C, 55.13; H, 5.14; N, 4.41; S, 15.24. ^{1}H NMR (400 MHz, $CDCl_3$) δ (ppm): 7.11–7.04 (br, 6H), 4.40–4.35 (br, 4H), 3.48–3.38 (br, 40H), 3.25–3.14 (br, 12H), 2.43–2.40 (br, 2H).

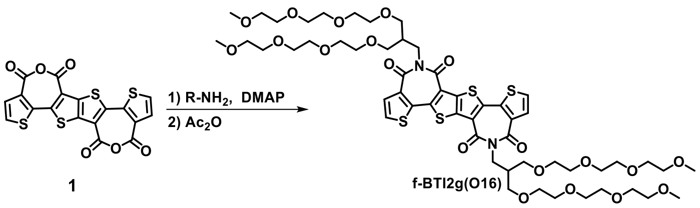

Synthesis of compound ***f-BTI2g(O16)***

4-Dimethylaminopyridine (DMAP, 333 mg, 2.73 mmol) and R-NH$_2$ (0.815 mg, 2.05 mmol) were added to a suspension of compound **1** (303 mg, 0.68 mmol) in 20 mL anhydrous 1,4-dioxane. The reaction mixture was stirred and heated to 80 °C overnight. After cooled to room temperature, 20 mL acetic anhydride was added via syringe in one portion. The reaction was further heated to 80 °C for 6 h. After cooled to room temperature, the reaction mixture was extracted with CH_2Cl_2 three times and the combined organic layer was dried over Na_2SO_4. The solvent was removed under a reduced pressure to afford a residue, which was purified by column chromatography over silica gel using dichloromethane:methanol (50:1) as the eluent to give f-BTI2g(O16) (0.70 g, yield: 86%). ^{1}H NMR (400 MHz, $CDCl_3$) δ (ppm): 7.80-7.79 (d, *J* = 10.6 Hz, 2H), 7.35-7.34 (d, *J* = 10.6 Hz, 2H), 4.45-4.43 (d, *J* = 13.9 Hz, 4H), 3.62-3.50 (m, 54H), 3.36 (s, 12H), 2.52-2.49 (m, 2H). ^{13}C NMR (100 MHz, $CDCl_3$) δ(ppm): 160.79, 160.66, 141.49, 137.97, 137.81, 133.87, 133.10, 125.54, 123.81, 71.91, 70.63, 70.59, 70.50, 70.23, 67.82, 59.03, 44.15.

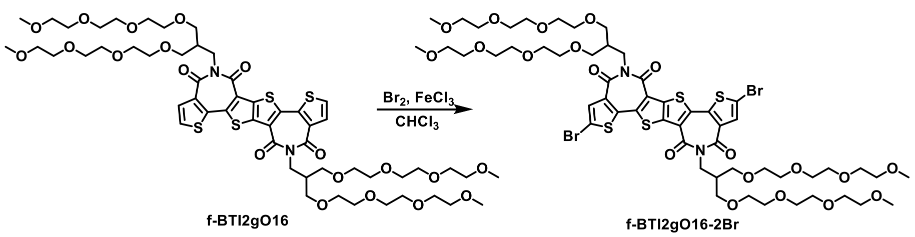

Synthesis of monomer ***f-BTI2g-2Br(O16)***

Br_2 (0.13 g, 0.78 mmol) in 2 mL chloroform was added dropwise into a solution of compound f-BTI2g(O16) (0.37 g, 0.31 mmol) and $FeCl_3$ (0.005 g) in 10 mL chloroform at 0 °C. The reaction was warmed to room temperature and stirred overnight. 50 mL water was then

added, and the mixture was extracted with CH_2Cl_2 for 3 times. The organic solvent was removed under a reduced pressure. The residue was further purified by silica column chromatography using dichloromethane:methanol (40:1) as the eluent to give f-BTI2g-2Br(O16) as a yellow solid (0. 36g, yield 85%). Anal. Calcd. for $C_{54}H_{76}Br_2N_2O_{20}S_4$ (%): C, 47.65; H, 5.63; N, 2.06; S, 9.42. Found (%): C, 47.39; H, 5.87; N, 2.32; S, 9.31. ^{1}H NMR (400 MHz, $CDCl_3$) δ (ppm): 7.79 (s, 2H), 4.44-4.43 (d, J = 13.9 Hz, 4H), 3.60-3.46 (m, 54H), 3.36 (s, 12H), 2.53-2.48 (m, 2H). ^{13}C NMR (100 MHz, CDCl3) δ (ppm): 160.83, 160.11, 140.16, 139.14, 138.15, 136.20, 133.61, 124.31, 113.63, 71.90, 70.95, 70. 59, 70.58, 70.49, 70.40, 59.04, 45.96, 38.14.

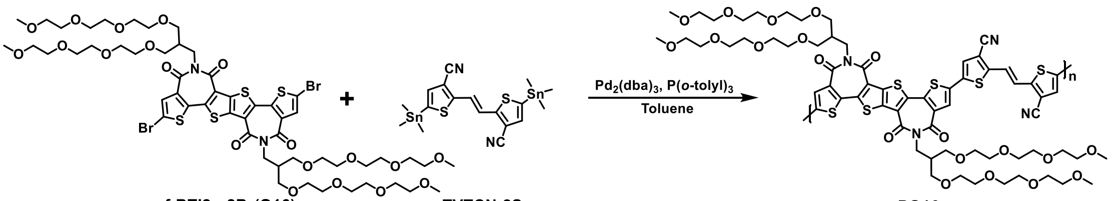

f-BTI2g-2Br(O16) TVTCN-2Sn PO16

*Synthesis of polymer **PO16***

To a 5 mL vial was charged with monomer f-BTI2g-2Br(O16) (0.14 g, 0.1 mmol), 2,5-bis(trimethylstannyl)thiophene (TVTCN-2Sn) (0.057 g, 0.1 mmol), tris(dibenzylideneacetone) dipalladium(0) ($Pd_2(dba)_3$, 4.0 mg), and tris(*o*-tolyl)phosphine (P(*o*-tolyl)$_3$, 10 mg). The vial was subjected to 3 pump/purge cycles with argon before anhydrous toluene (3 mL) was added. The vial was sealed under argon flow and then heated at 140 ºC by microwave irradiation for 3 h. After cooled down to room temperature, the reaction mixture was dripped into 100 mL methanol. The precipitated solid was filtrated and further purified by Soxhlet extraction with methanol, acetone, hexane, dichloromethane, and chloroform, sequentially. The final chloroform fraction was concentrated and dripped into methanol. The precipitate was collected and dried to afford product polymer PO16 (0.12 g, yield: 85%) as a black solid. Anal. calcd. for $C_{66}H_{80}N_4O_{20}S_6$ (%): C, 54.98; H, 5.59; N, 3.89; S, 13.34. Found (%): C, 55.03; H, 5.47; N,

3.91; S, 13.54. ^{1}H NMR (400 MHz, $CDCl_3$) δ (ppm): 7.42-7.25 (br, 6H), 4.49–4.27 (br, 4H), 3.52–3.41 (br, 54H), 3.26–3.14 (br, 12H), 2.44–2.38 (br, 2H).

2. Materials Characterization

(1) Basic properties characterization

^{1}H NMR spectra were recorded on Bruker Ascend-400 spectrometer. The chemical shifts were reported in ppm relative to the tetramethylsilane (TMS) internal standard. The elemental analysis of monomers and polymers were conducted on a Vario EL CUBE elemental analyzer. Molecular weights of the polymers were determined by Polymer Laboratories PL-GPC 50 system. The GPC data were measured using two PLgel MIXED-B LS (300 × 7.5mm) columns at 35 °C with a flow rate of 1 mL min^{-1} and polymethyl methacrylate (PMMA) as standard. Thermogravimetric analysis (TGA) and differential scanning calorimetry (DSC) curves of the polymers were recorded on a Mettler STARe TA Instrument and a Mettler STARe DSC System, respectively, both at a heating/cooling ramp of 10 ºC min^{-1} under a N_2 flow rate of 90 mL min^{-1}. UV-vis absorption spectra were recorded on a Shimadzu UV-3600 UV/vis/NIR spectrophotometer. To obtain the LUMO/HOMO energy levels of the polymers, cyclic voltammetry (CV) measurements were carried out on a CHI660 Potentiostat/Galvanostat electrochemical workstation at a scan rate of 50 mV s^{-1} using 0.1 M tetra(*n*-butyl)ammonium hexafluorophosphate in acetonitrile as the supporting electrolyte, a platinum disk as the working electrode, a platinum mesh as the counter electrode, and Ag/Ag^+ as the reference electrode. For these CV measurements, ferrocene/ferrocenium (Fc/Fc^+) redox couple was used as the external reference. To evaluate the electrochemical properties of the polymer films in aqueous solution, the films were casted onto indium tin oxide (ITO) glass substrates (resistivity: 10–15 ohm sq^{-1}) as work electrode, their CV curves were then collected by the same three electrodes setup for standard CV using 0.1 M NaCl supporting electrolyte at a scan rate of 50 mV s^{-1}. Two-dimensional grazing incidence wide angle X-ray scattering (2D-GIWAXS) measurements were

performed at the Beamline PLS-II 9A SAXS of Pohang Accelerator Laboratory, Republic of Korea.

ESR measurements were performed on an X-band (9.84 GHz) Burker EMXplus-10 spectrometer, using a microwave power of 2 mW, a magnetic field modulation magnitude of 2 gausses and a modulation frequency of 100 kHz, respectively. The polymer films were prepared on PET substrates and cut into 3 mm by 12 mm in size, and then loaded into tightly sealed quartz ESR tubes. The spin-counting was done with Burker software following standard procedures. AFM measurements of polymer films were conducted using a Dimension Icon Scanning Probe Microscope (Asylum Research, MFP-3D-Stand Alone) in tapping mode.

(2) OTFT fabrication and characterization

Borosilicate glass substrates were diced into 1.2 cm × 1.2 cm after cleaning process. For spin-coated devices, source-drain electrodes (3 nm Cr and 30 nm Au) with a channel length (L) of 10, 20, 50, 100 μm and a channel length width (W) of 5000 μm were patterned by photolithography. Next, the patterned glass substrates were cleaned by sonication in acetone and isopropanol for 15 min each, followed by UV-ozone treatment for 15 min. After that, the substrates were transferred into the N_2-filled glove box (O_2, H_2O concentration < 0.1 ppm). The polymer active layer was spin-coated from 60 °C chloroform solution (5 mg mL^{-1}) followed by a 10 min thermally annealing at 130 °C (thickness: ~30 nm). After 10 min cooling-down process, diluted CYTOP solution (CTL-809M:CT-SOLV180 = 2:1, volume ratio, Asahi Glass Co., Ltd.) was spin-coated on the active layer with a 400 nm thickness, then annealed at 90 °C for 30 min. Finally, 50 nm Al was thermally evaporated on top as the gate electrode.

The devices were characterized by Keithley 4200-SCS semiconductor analyzer inside a N_2-filled glove box. For the transfer curves, drain current (I_D) was measured as V_G (gate voltage) was swept keeping a constant source to drain voltage (V_{SD}) of 5 V and 80 V. For the output curves, a variety of gate voltages (V_Gs) were fixed with a 10 V step and V_{SD} was swept from 0 to 80 V. The mobilities in linear and saturation regimes were extracted from the equation:

$$I_{\mathrm{SD(lin)}} = \frac{W}{L}\mu C_{\mathrm{i}}\left(V_{\mathrm{SG}} - V_{\mathrm{T}} - \frac{V_{\mathrm{SD}}}{2}\right)V_{\mathrm{SD}} \quad (1)$$

$$I_{\mathrm{SD(sat)}} = \frac{W}{2\mathrm{L}}\mu C_{\mathrm{i}}(V_{\mathrm{SG}} - V_{\mathrm{T}})^2 \quad (2)$$

Where L is the channel length, W is the channel width, C_i is the capacitance per unit area of the insulating layer, and V_T is the threshold voltage.

(3) OTE fabrication and characterization

Borosilicate glass substrates were cleaned by sonication washing using acetone and isopropanol as the solvents sequentially for 15 min each, followed by 15 min UV-ozone treatment. 30 nm Au electrodes of 100 μm length and 2000 μm width were deposited by shadow mask through thermal evaporation. Next, 0.1 nm Au particles were thermally evaporated on the substrate. The polymer film was spin-coated from solution (4 mg mL^{-1} in chloroform for PO8; 4 mg mL^{-1} in chloroform for PO16; 5 mg mL^{-1} in hexafluoroisopropanol for PO12) at 1500 rpm for 60 s and annealed at 220 130, 220 °C, respectively for 10 min (thickness: ~25 nm for PO8, ~55 nm for PO12, and ~39 nm for PO16). Then *N*-DMBI solution (0.1-5 mg mL^{-1} in *n*-butyl acetate) was spin-coated on top at 5000 rpm for 10 s followed by thermal annealing at 120 °C for 10 s to activate the doping. For electrical conductivity measurement, the linear *I*-*V* curves were characterized by Keithley 4200-SCS system in a N_2-filled glove box. According to Ohm's layer, average electrical conductivity values were calculated from five different measurements. For thermoelectric performance measurement, using a pair of Peltier devices with a separation distance of 1 mm, a temperature difference (ΔT) was applied between the electrodes by a pair of thermal couples. During the measurement, the temperature difference (ΔT) ranged from 0 to 2.8 K with a 0.7 K step. Then the thermal voltage records responded by Keithley 4200-SCS system in a N_2-filled glove box were used to extract Seebeck coefficients through the linear fitting of the thermal voltage versus ΔT. All the Seebeck coefficients were the average values from five different measurements.

(4) Hall effect measurement

An AC-field Hall protocol was employed to investigate carrier concentration and mobility for each polymer film. The samples were measured using the AC field Hall method with a Lake Shore model 8404 system with an AC-field amplitude of 1.2T RMS. The detailed fabrication information of sample followed the literature of Pei and co-workers.[21]

3. Supplementary Tables

Table S1. Summary of n-type polymers-based OTE performance parameters reported in literature.

Polymers	μ_{OTFT} (cm^2 V^{-1} s^{-1})	Dopant	σ (S cm^{-1})	*S* (mV K^{-1})	*PF* (μW m^{-1} K^{-2})	Year	Ref.
(P(NDI2OD-T2)	NA	N-DMBI	8×10^{-3}	−850	6×10^{-7}	2014	2
(P(NDI2OD-T2)	NA	N-DMPI	4×10^{-3}	−770	6×10^{-7}	2014	2
PPV(BDPPV)	~1	N-DMBI	14	NA	28	2015	3
BBL	NA	TDAE	2.4	-60	0.43	2016	4
PNDTI-BBT-DT	3.3×10^{-3}	N-DMBI	0.18	-56	0.6	2017	5
PNDTI-BBT-DP	0.011	N-DMBI	5	-169	14.2	2017	5
p(gNDI-gT2)	NA	N-DMBI	0.3	-190	0.4	2018	6
N2200	7.2×10^{-3}	N-DMBI	0.001	-290	0.01	2018	7
TEG-N2200	NA	N-DMBI	0.17	-112	0.4	2018	7
P(NDI2OD-Tz2)	NA	TDAE	0.1	-477	1.5	2018	8
PDPH	1.70	N-DMBI	1×10^{-3}	NA	7×10^{-4}	2018	9
PDPF	1.51	N-DMBI	1.5	NA	4.65	2018	9
P(PzDPP-CT2)	0.79	N-DMBI	8.4	-380	9.3	2019	10
P(TDPP-CT2)	0.32	N-DMBI	0.39	-620	57.3	2019	10
PDTzTI	0.8	N-DMBI	4.6	-129	7.6	2019	11
LPPV-1	0.16	N-DMBI	1.1	−170	1.96	2019	12
LPPV-2	0.013	N-DMBI	0.07	NA	0.25	2019	12
pNDI-2T	NA	N-DMBI	0.013	-324	0.035	2020	13
UFBDPPV	NA	TAM	25	-198	80	2020	14
pNB-Tz	0.12	N-DMBI	0.87	-250	9.9	2020	15
pNB-TzDP	0.55	N-DMBI	11.6	-200	53.4	2020	15
PI-BN	NA	N-DMBI	1×10^{-3}	-454	0.02	2020	16
P1	0.01	N-DMBI	0.2	−22	0.12	2020	17
P2	7×10^{-3}	N-DMBI	0.28	−21	0.16	2020	17
P3	3×10^{-3}	N-DMBI	0.008	−210	0.034	2020	17
PCNTI	0.018	N-DMBI	0.19	-123	0.25	2021	18
PCNDTI	0.0018	N-DMBI	0.28	-73	0.1	2021	18
PCNI-BTI	0.13	N-DMBI	23.3	-83	10	2021	18
PCNDTI-BTI	0.0092	N-DMBI	0.56	-94	0.33	2021	18
BBL	NA	BVc+	1.63	-199	5.370.11	2021	19
BBL	NA	PEIlin	8	-146	12	2021	20
PNDI2TEG-2Tz	0.035	N-DMBI	1.36	-167	3.8	2021	21
PNDI2C8TEG-2Tz	0.048	N-DMBI	1.6	-326	16.5	2021	21
PNDI2TEG-T2DO	NA	N-DMBI	8×10^{-4}	−254	10^{-6}	2021	22
PNDI2OD-T2DEG	NA	N-DMBI	0.002	−247	10^{-5}	2021	22
PNDI2TEG-T2DEG	NA	N-DMBI	0.08	−290	10^{-5}	2021	22
PBDOPVTT	NA	N-DMBI	0.2	-1854	67	2021	23
TBDOPV-T	NA	N-DMBI	65	NA	106	2021	24
PBN-19	0.029	TADE	7.8	−179	24.8	2021	25
N-N	0.33	N-DMBI	0.65	−260	3.2	2021	26
A-N	0.23	N-DMBI	0.26	−231	1.6	2021	26
A-A	0.07	N-DMBI	0.018	−536	0.25	2021	26

p(g7NC2N)	NA	N-DMBI	4.8	NA	6.2	2022	27
p(g7NC4N)	NA	N-DMBI	8.0	NA	10.9	2022	27
p(g7NC6N)	NA	N-DMBI	1.0	NA	2.1	2022	27
p(g7NC8N)	NA	N-DMBI	1.25	NA	3.1	2022	27
p(g7NC10N)	NA	N-DMBI	1.81	NA	5.22	2022	27
p(g7NC12N)	NA	N-DMBI	0.88	NA	1.35	2022	27
p(g7NC16N)	NA	N-DMBI	0.31	NA	0.94	2022	27
PDTzTI-TEG	NA	N-DMBI	34	−170	15.7	2022	28
PO8	NA	N-DMBI	18.1	−110.2	14.7	This work	
PO12	0.013	N-DMBI	92.0	−162.6	94.3		
PO16	0.008	N-DMBI	43.5	−95.4	62.1		

Table 2. Optical properties of the monomers f-BTI2g-Br(O8), f-BTI2g-Br(O12), and f-BTI2g-Br(O16).

Monomer	λ_{max}^{soln} [nm] [a]	λ_{max}^{film} [nm] [b]	E_g^{opt} [eV] [c]
f-BTI2g-Br(O8)	461	450	2.40
f-BTI2g-Br(O12)	461	450	2.40
f-BTI2g-Br(O16)	461	450	2.40

[a] Absorption of solution (10^{-5} M in chlorobenzene). [b] Absorption of as-cast film from 5 mg mL^{-1} chlorobenzene solution. [c] Optical bandgap (E_g^{opt}) derived from absorption onset of polymer film using the equation: $E_g^{opt} = 1240/\lambda_{onset}^{film}$ (eV).

Table S3. Summary of the values of the carrier concentration (n) and mobility ($\mu_{e,doping}$) from AC magnetic field Hall effect measurements on three films with optimized N-DMBI concentrations.

Polymer	n (10^{20} cm^3)	μ (cm^2 V^{-1} s^{-1})
PO8	6.3 (6.18±0.1)	0.19 (0.18±0.2)
PO12	9.09 (9.04±0.4)	0.69 (0.64±0.3)
PO16	8.41 (8.30±0.1)	0.35 (0.33±0.2)

4. Supplementary Figures

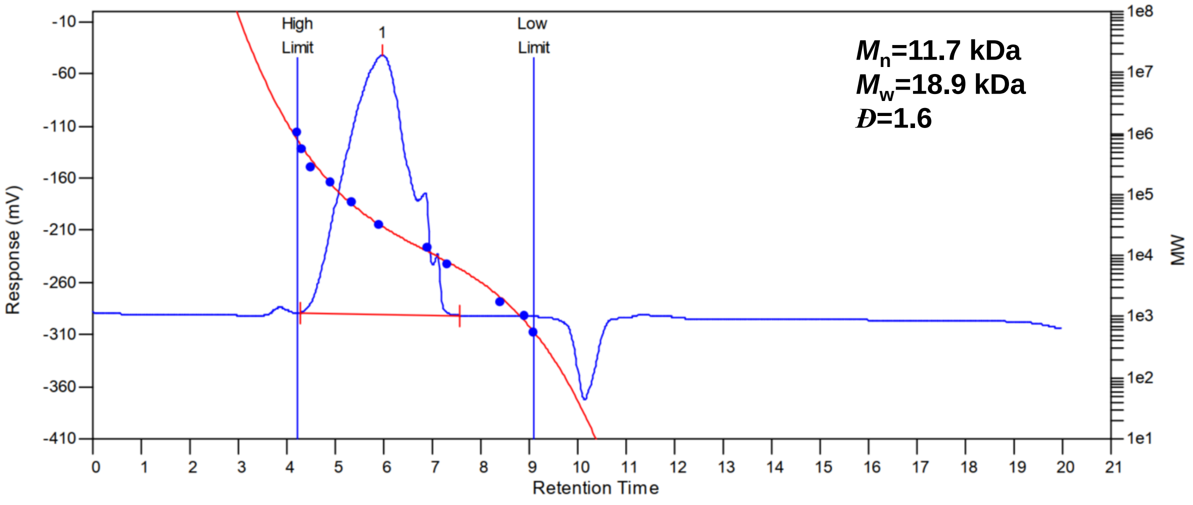

Figure S1. GPC curve of polymer PO8.

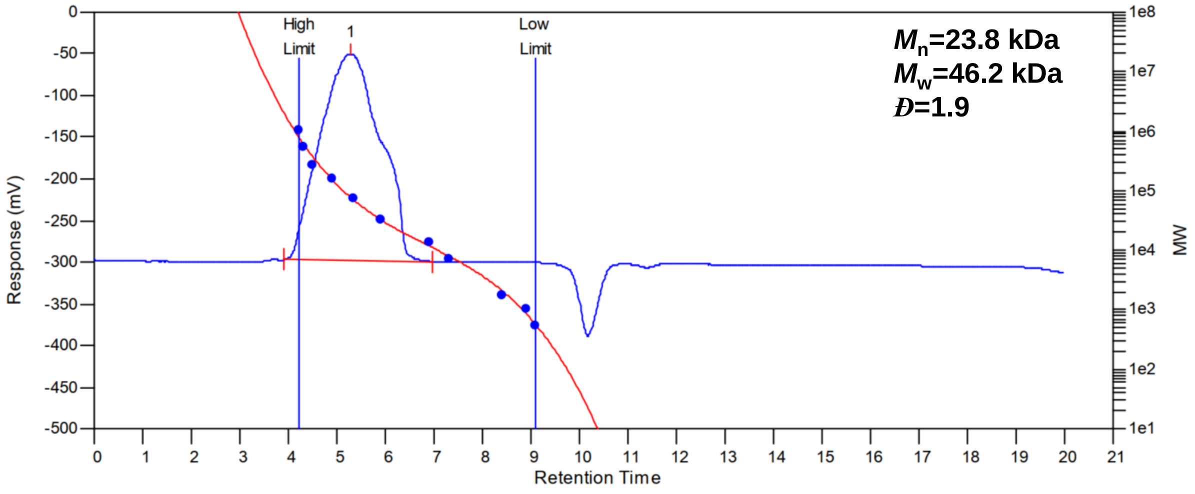

Figure S2. GPC curve of polymer PO12.

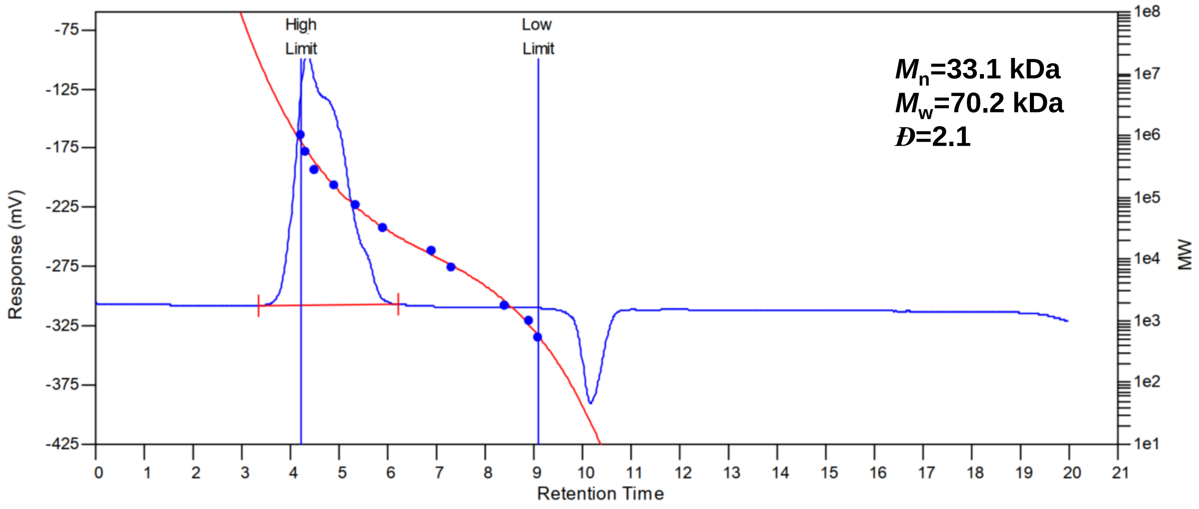

Figure S3. GPC curve of polymer PO16.

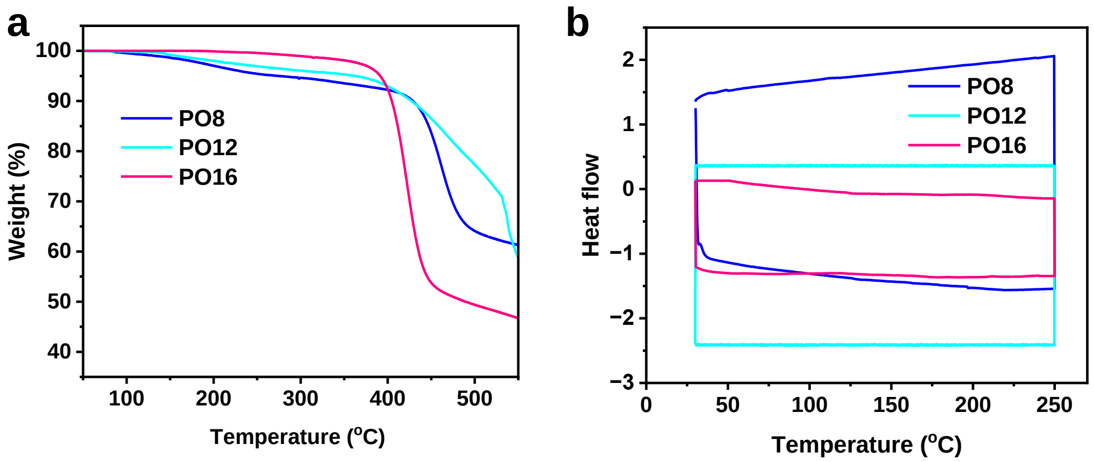

Figure S4. (a) Thermogravimetric (TGA) and (b) differential scanning calorimetry (DSC) curves of the polymers.

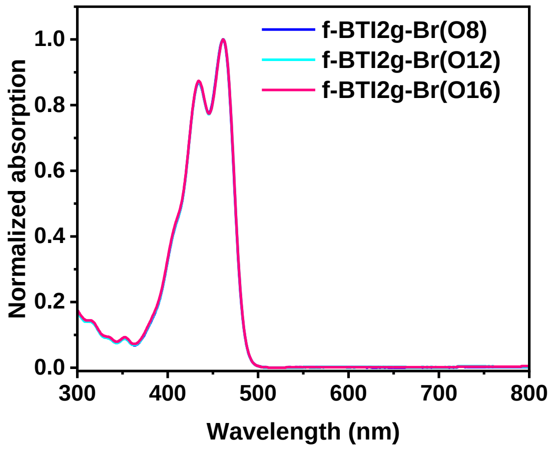

Figure S5. Normalized UV−vis absorption spectra of the dibrominated monomers in diluted chloroform solution (10^{-5} M).

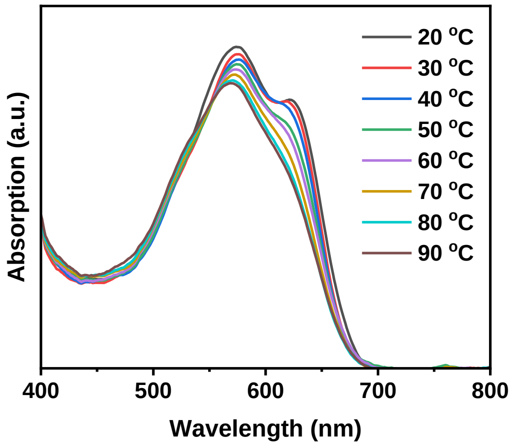

Figure S6. Variable-temperature UV-vis absorption spectra of PO8 in diluted chlorobenzene solutions at indicated temperatures.

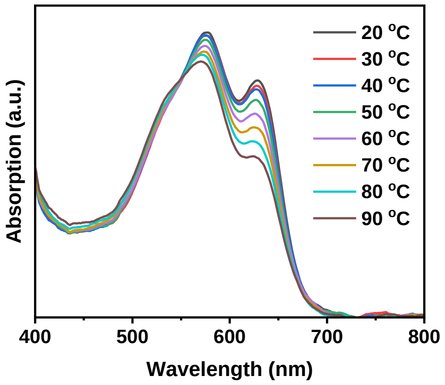

Figure S7. Variable-temperature UV-vis absorption spectra of PO12 in diluted chlorobenzene solutions at indicated temperatures.

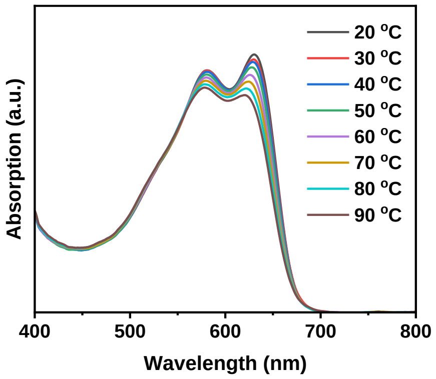

Figure S8. Variable-temperature UV-vis absorption spectra of PO16 in diluted chlorobenzene solutions at indicated temperatures.

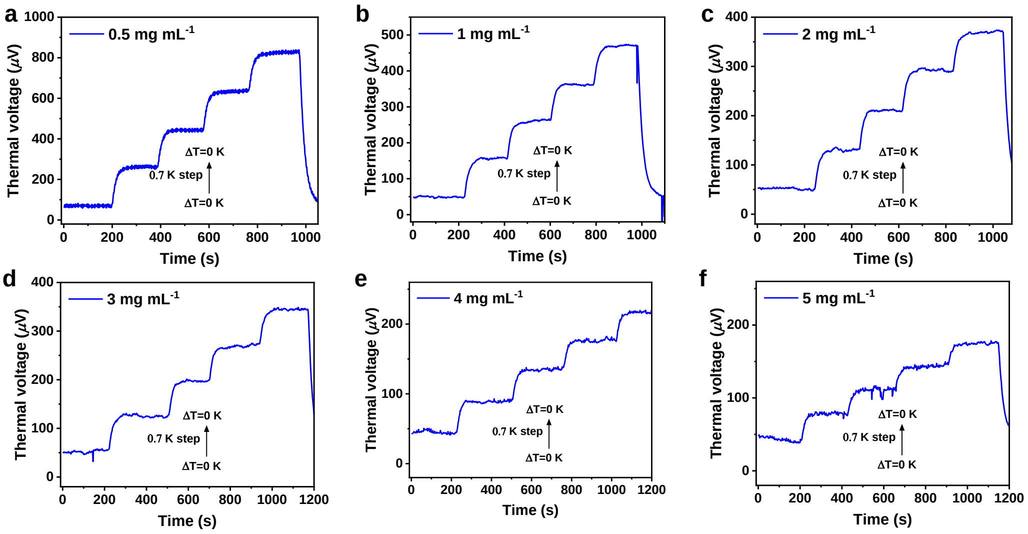

Figure S9. Representative thermal voltage response curves for sequentially doped PO8 polymer films with distinct N-DMBI concentrations in *n*-butyl acetate solutions, including (a) 0.1 mg mL^{-1}, (b) 0.5 mg mL^{-1}, (c) 1 mg ml^{-1}, (d) 2 mg mL^{-1}, (e) 3 mg mL^{-1}, (f) 4 mg mL^{-1}, (g) 5 mg mL^{-1}. During the measurement, the temperature difference (ΔT) across the doped polymer films was systematically varied from 0 to 2.8 K with a step of 0.7 K then back to 0 K.

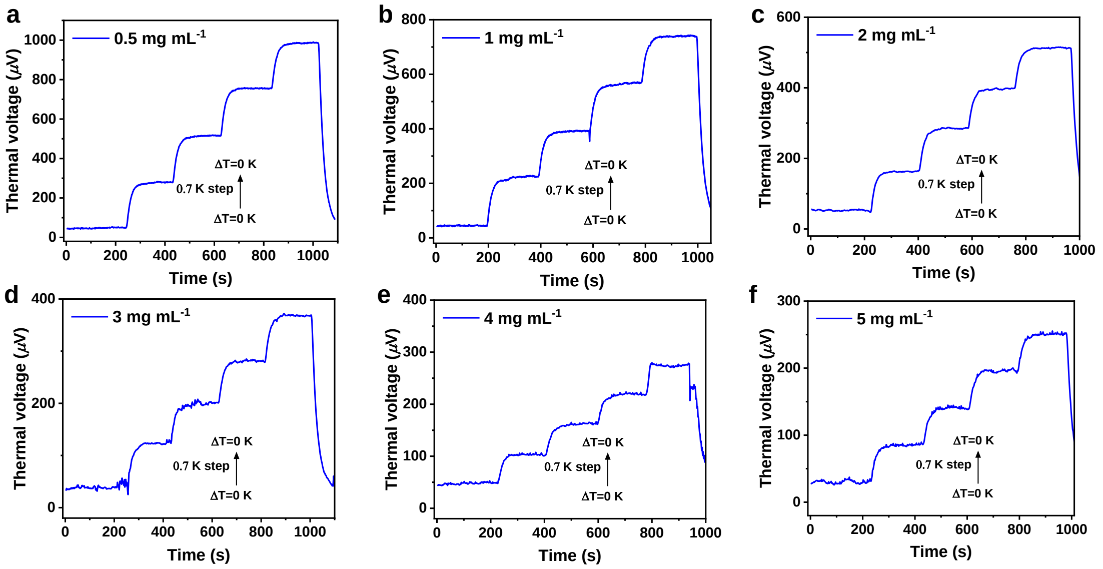

Figure S10. Representative thermal voltage response curves for sequentially doped PO12 polymer films with distinct N-DMBI concentrations in *n*-butyl acetate solutions, including (a) 0.1 mg mL^{-1}, (b) 0.5 mg mL^{-1}, (c) 1 mg ml^{-1}, (d) 2 mg mL^{-1}, (e) 3 mg mL^{-1}, (f) 4 mg mL^{-1}, (g) 5 mg mL^{-1}. During the measurement, the temperature difference (ΔT) across the doped polymer films was systematically varied from 0 to 2.8 K with a step of 0.7 K then back to 0 K.

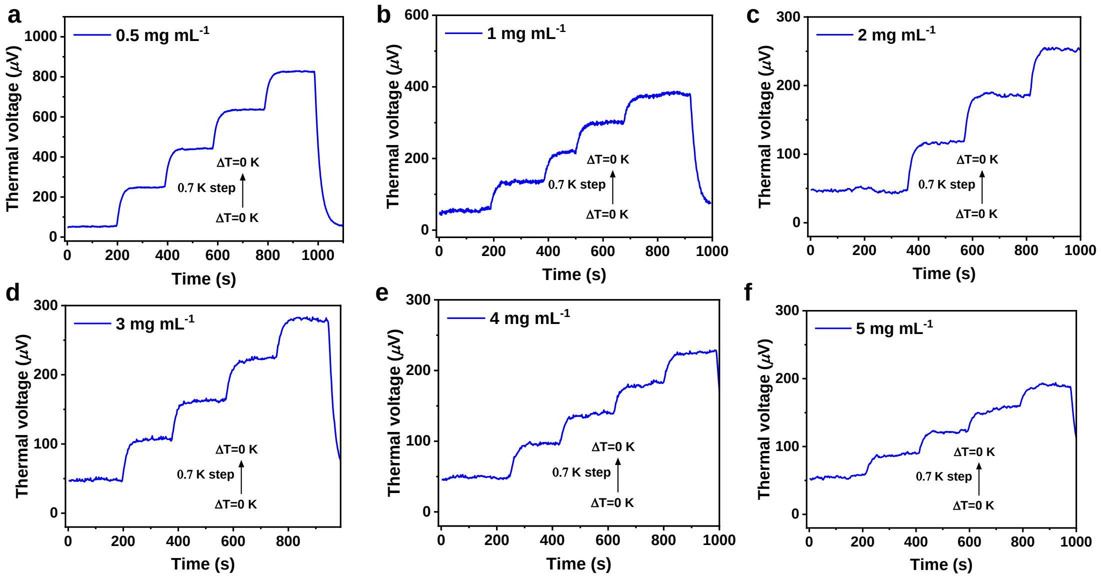

Figure S11. Representative thermal voltage response curves for sequentially doped PO16 polymer films with distinct N-DMBI concentrations in *n*-butyl acetate solutions, including (a) 0.1 mg mL^{-1}, (b) 0.5 mg mL^{-1}, (c) 1 mg ml^{-1}, (d) 2 mg mL^{-1}, (e) 3 mg mL^{-1}, (f) 4 mg mL^{-1}, (g) 5 mg mL^{-1}. During the measurement, the temperature difference (ΔT) across the doped polymer films was systematically varied from 0 to 2.8 K with a step of 0.7 K then back to 0 K.

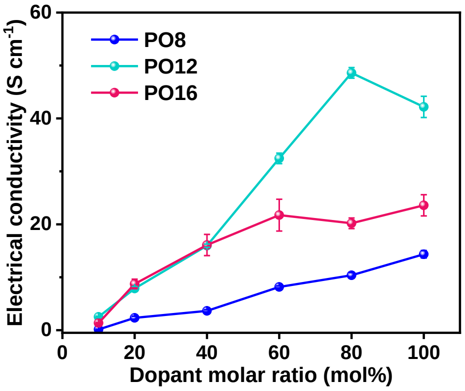

Figure S12. The average electrical conductivity of blending-doped three films as function of dopant ratio.

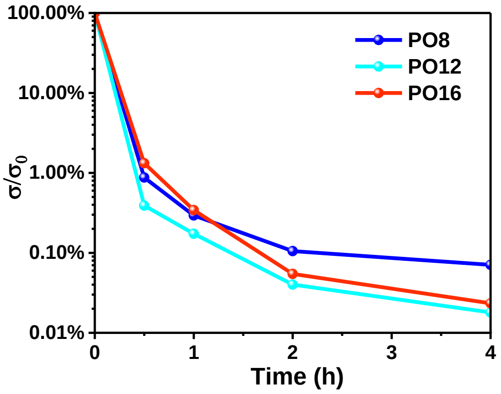

Figure S13 Temporal evolution of the electrical conductivity performance of the doped polymer films in ambient environment.

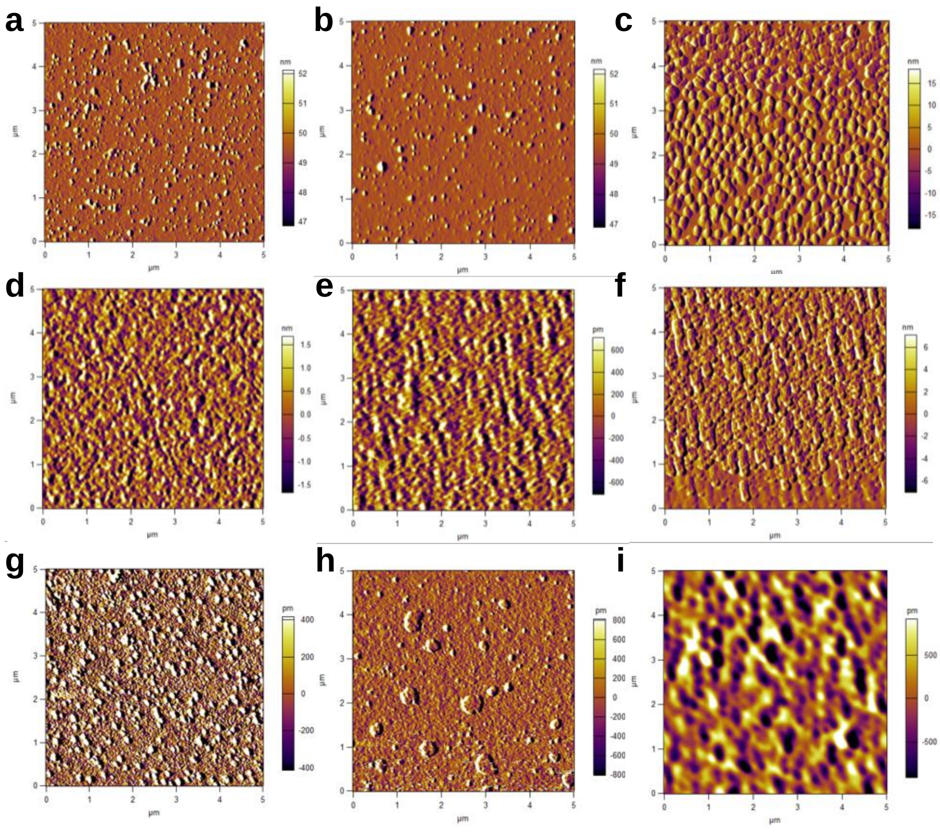

Figure S14. AFM phase images of (a, b, c) PO8, (d, e, f) PO12, and (g, h, i) PO16 films: as-cast (a. d, g), annealed (b, e, h) and (c, f, i) n-doped by *N*-DMBI.

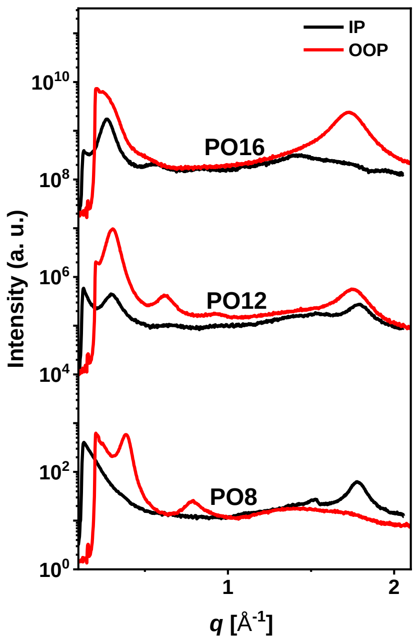

Figure S15. The in-plane and out-of-plane line-cut profiles of the 2D GIWAXS images of pristine polymer films.

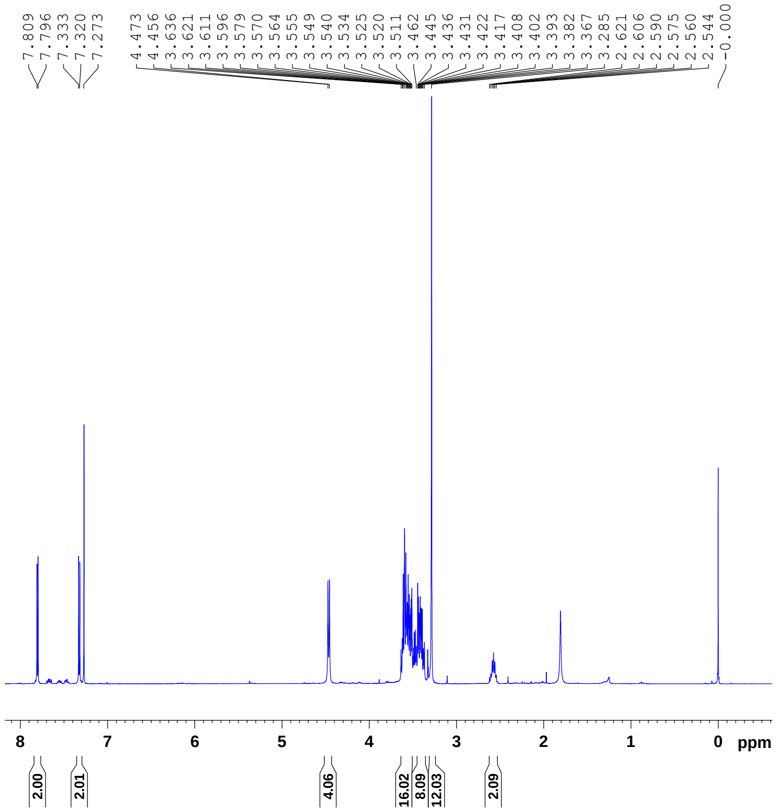

Figure S16. The ^{1}H NMR spectrum of f-BTI2g(O8).

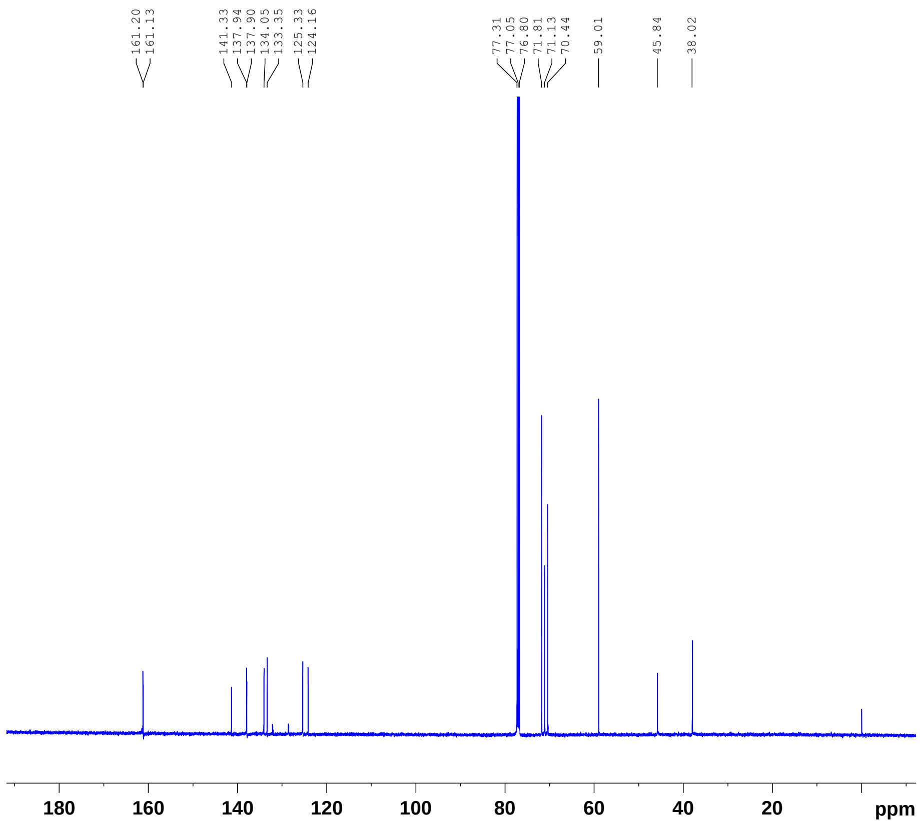

Figure S17. The ^{13}C NMR spectrum of f-BTI2g(O8).

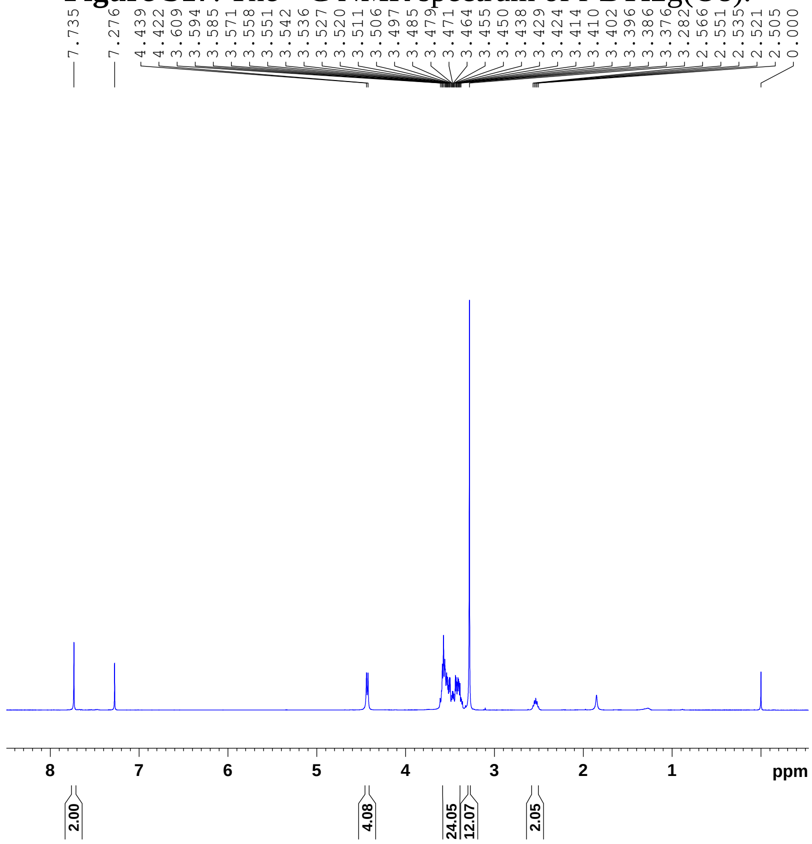

Figure S18. The ^{1}H NMR spectrum of f-BTI2g-2Br(O8).

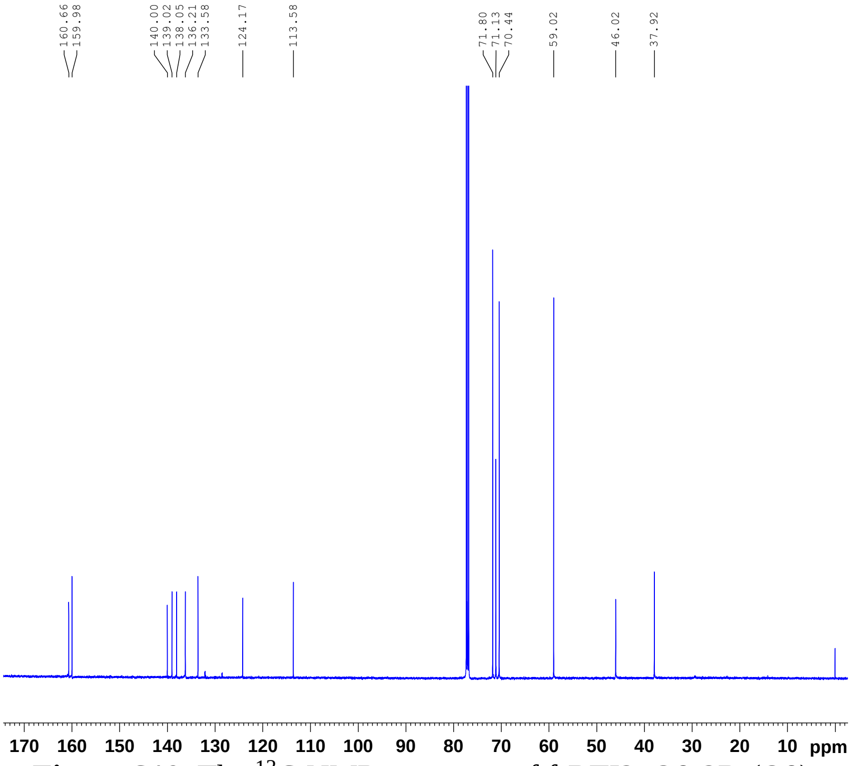

Figure S19. The ^{13}C NMR spectrum of f-BTI2gO8-2Br(O8).

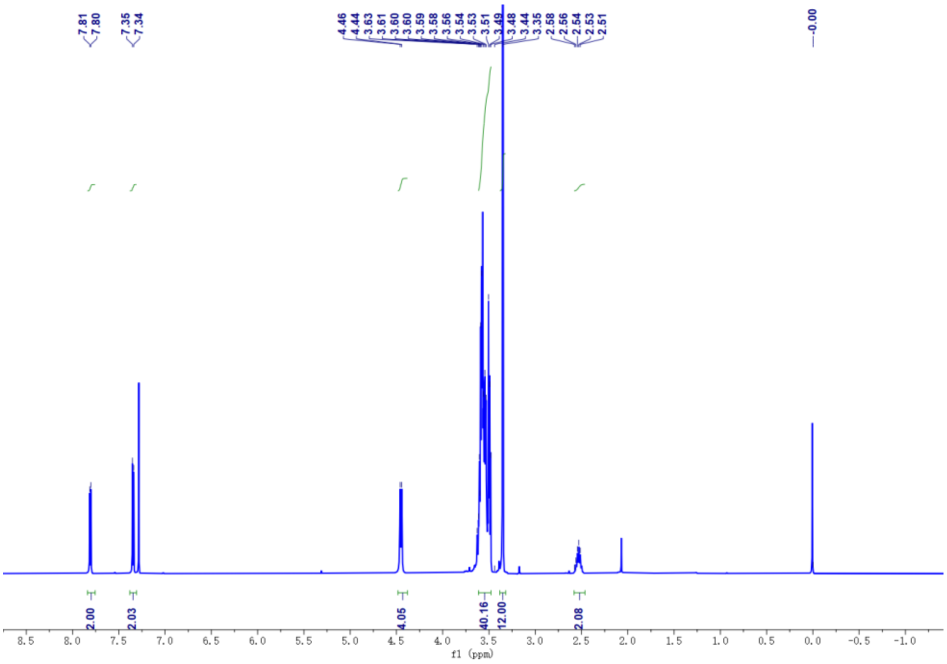

Figure S20. The ^{1}H NMR spectrum of f-BTI2g(O12).

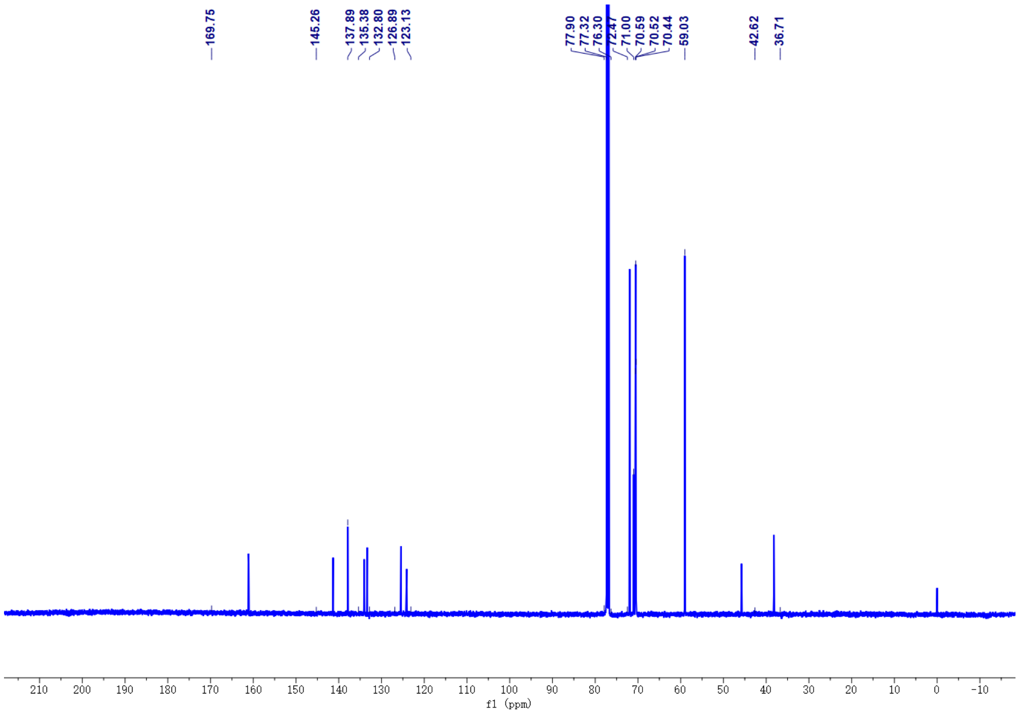

Figure S21. The ^{13}C NMR spectrum of f-BTI2g(O12).

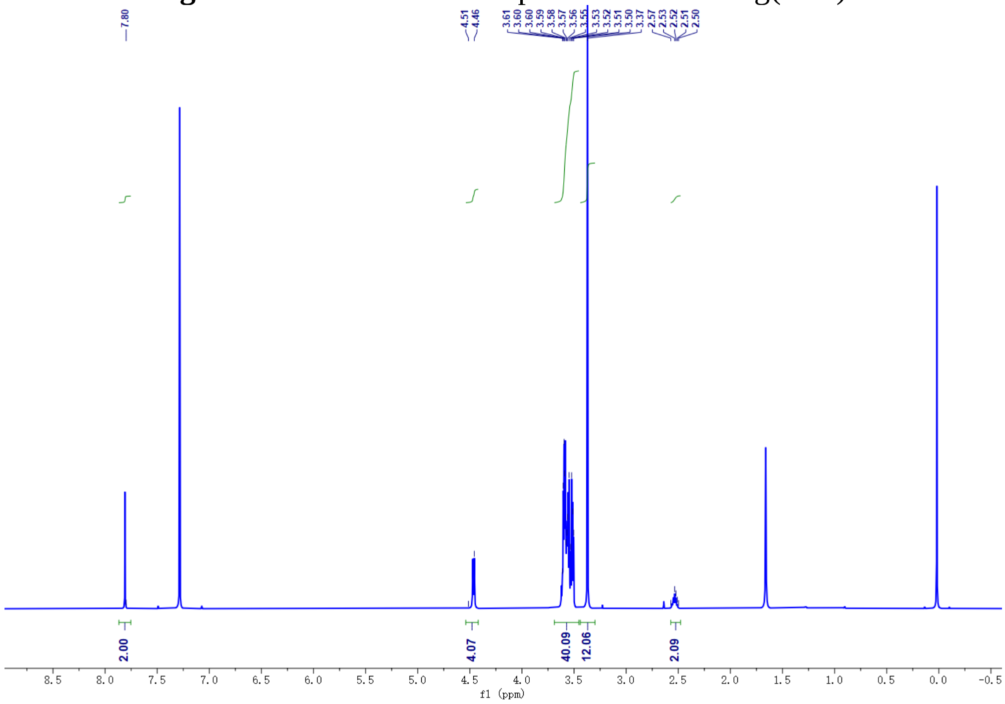

Figure S22. The ^{1}H NMR spectrum of f-BTI2g-2Br(O12).

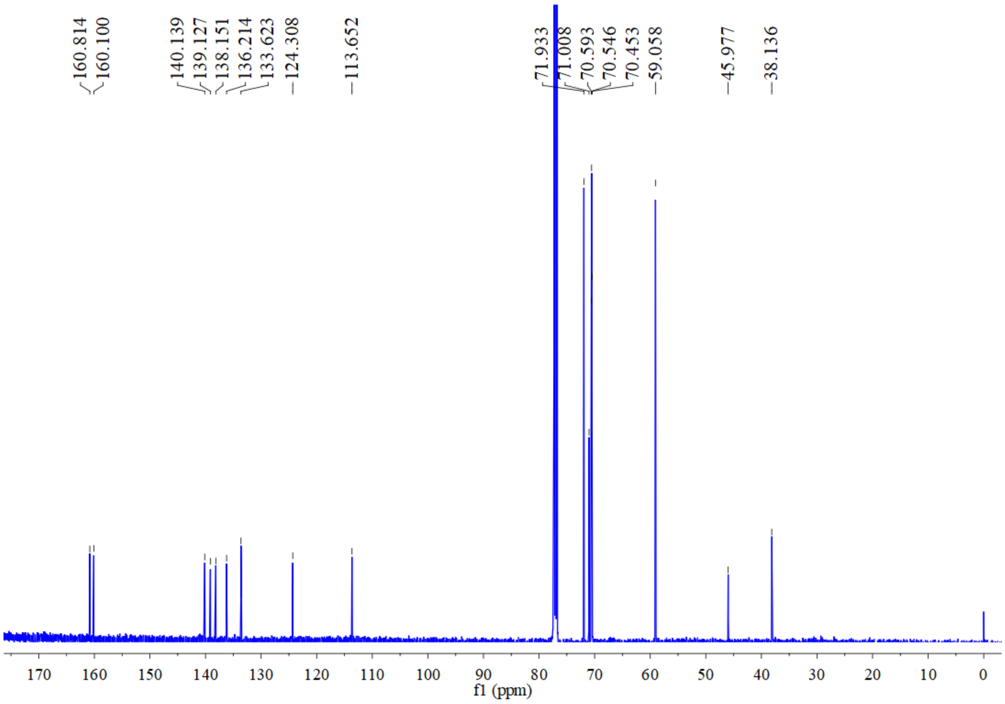

Figure S23. The ^{13}C NMR spectrum of f-BTI2g-2Br(O12).

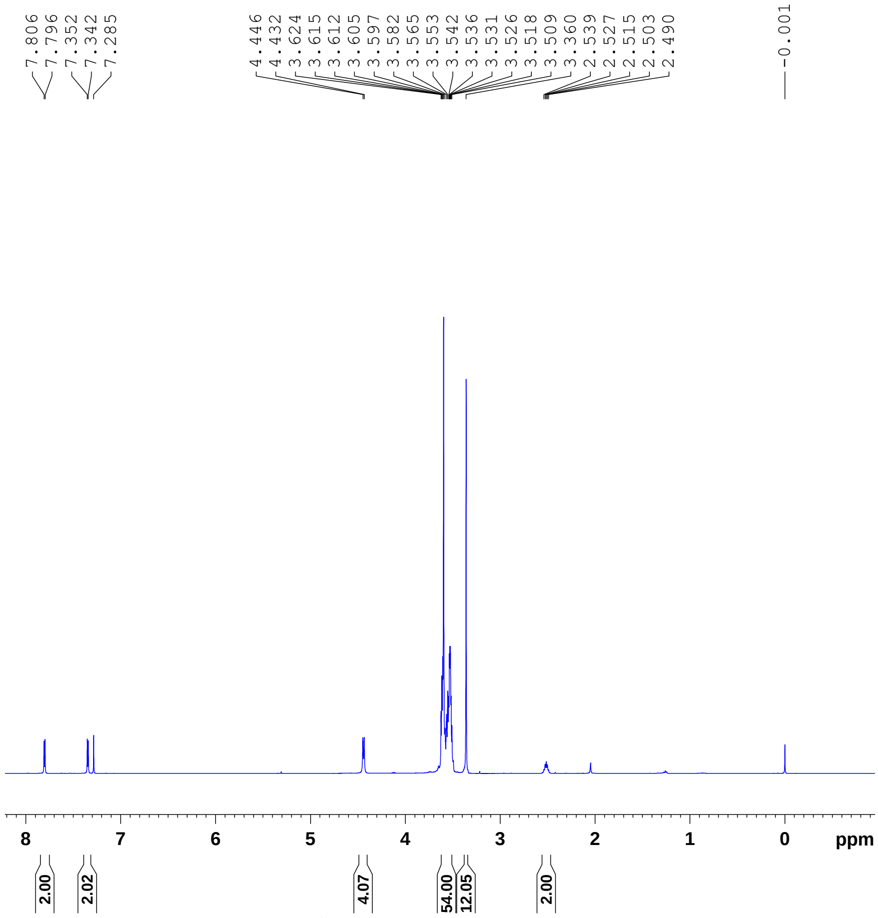

Figure S24. The ^{1}H NMR spectrum of f-BTI2g(O16).

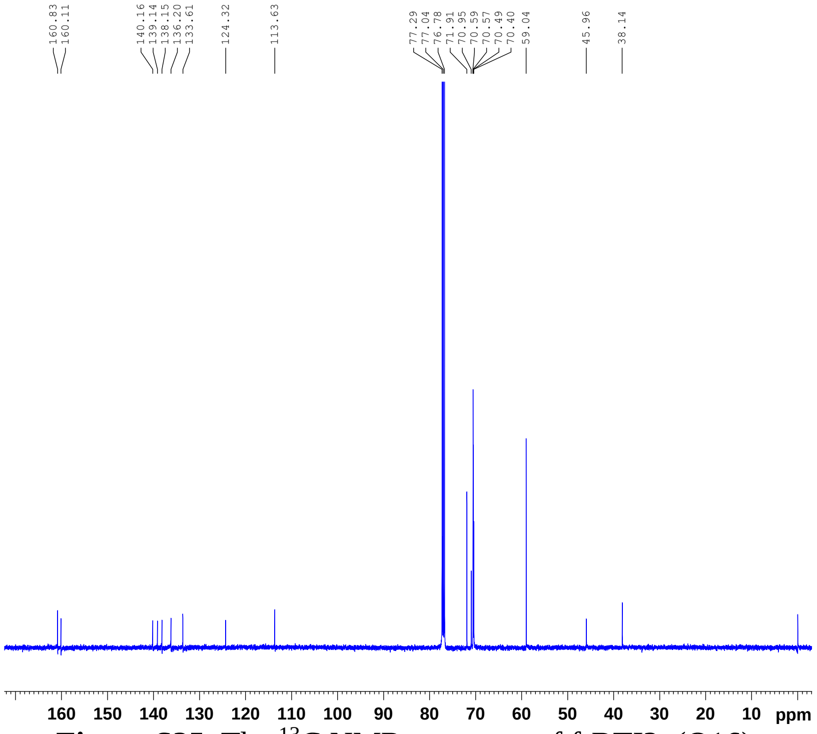

Figure S25. The ^{13}C NMR spectrum of f-BTI2g(O16).

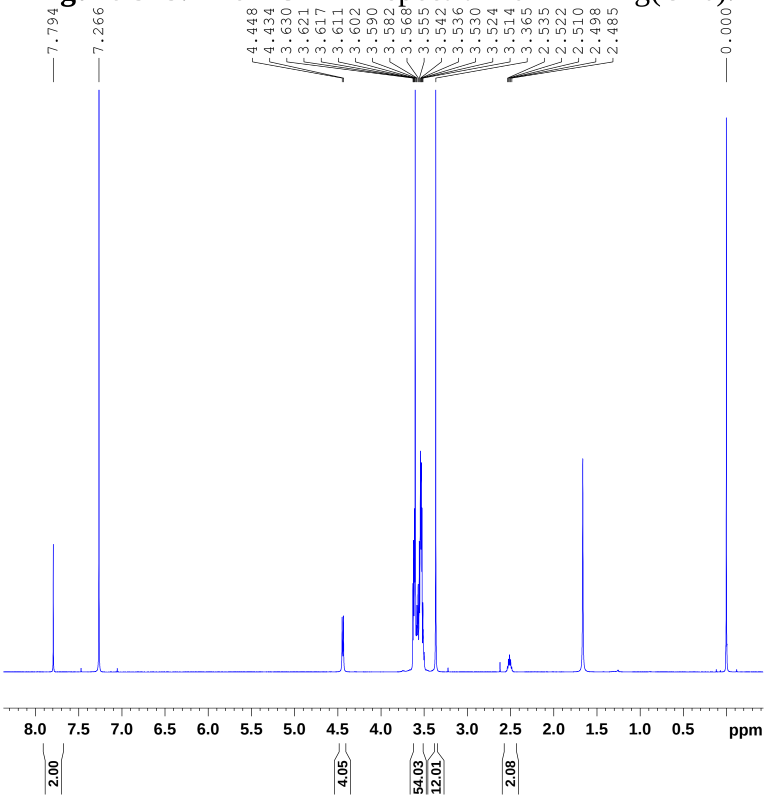

Figure S26. The ^{1}H NMR spectrum of f-BTI2g-2Br(O16).

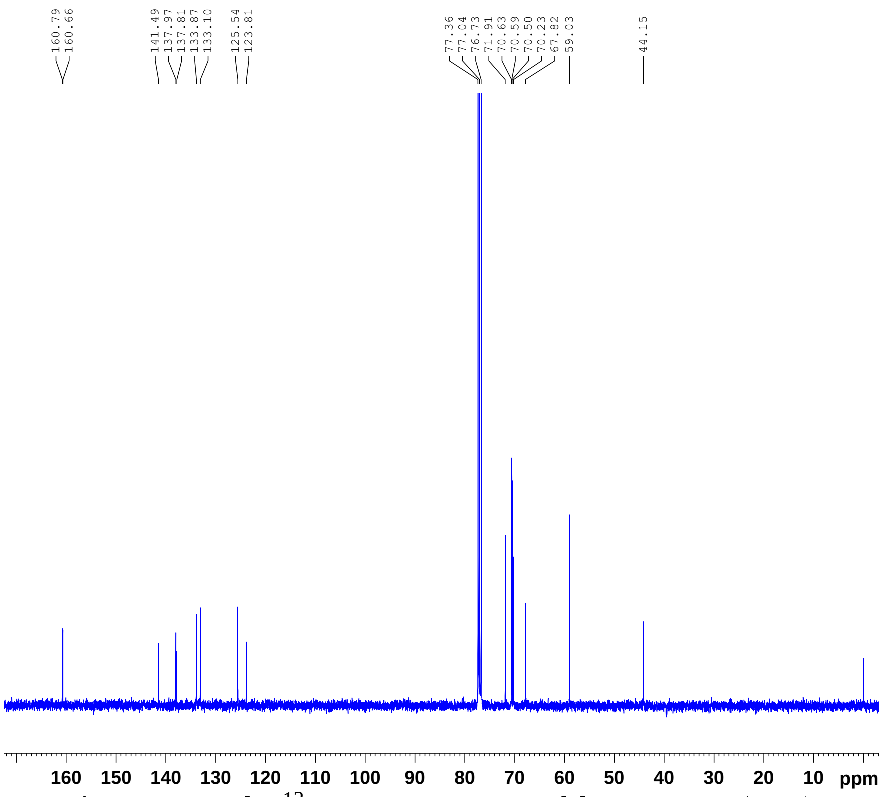

Figure S27. The ^{13}C NMR spectrum of f-BTI2g-2Br(O16).

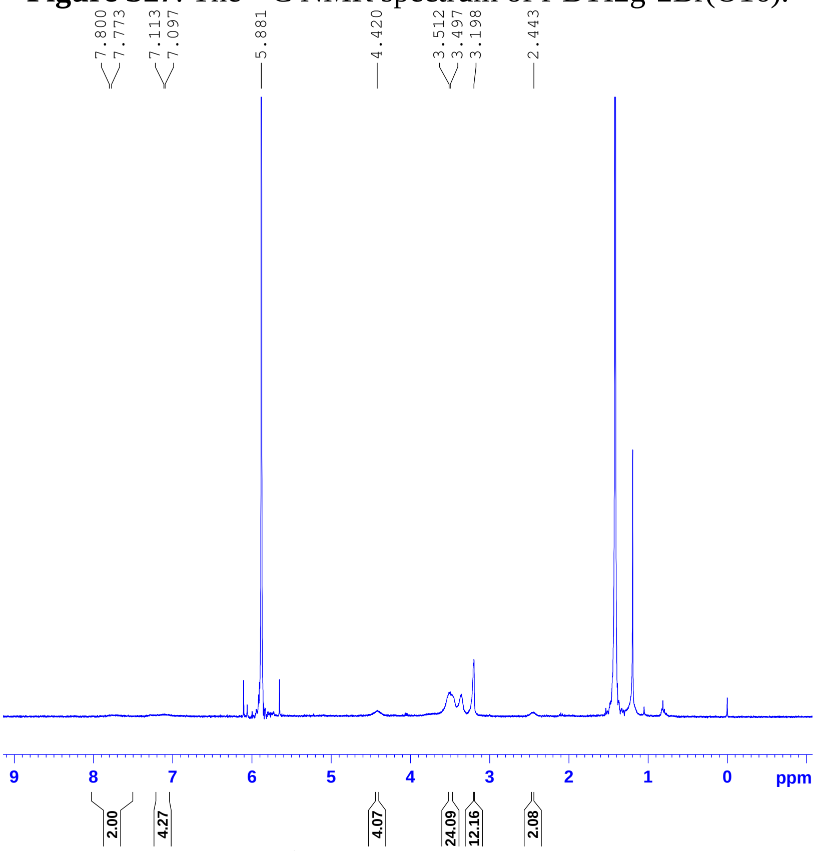

Figure S28. The ^{1}H NMR spectrum of polymer PO8.

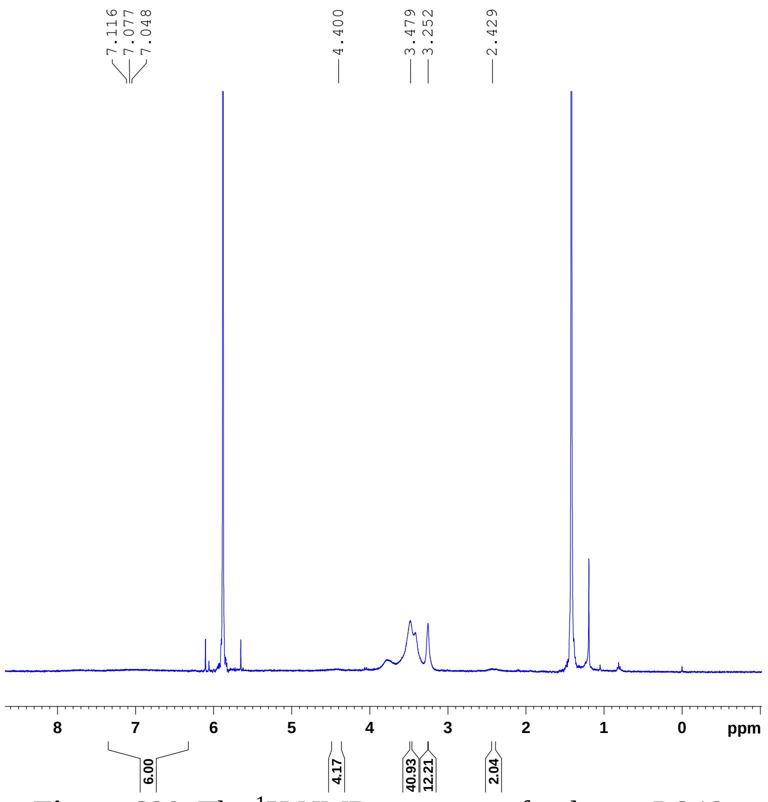

Figure S29. The ^{1}H NMR spectrum of polymer PO12.

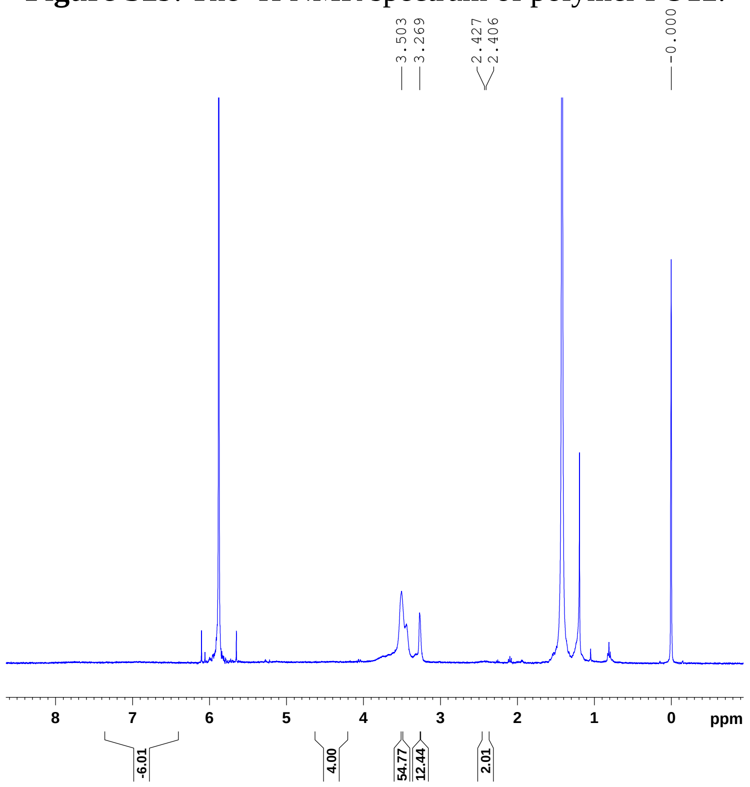

Figure S30. The ^{1}H NMR spectrum of polymer PO16.

References

1. D. Kim, H. Zhu, A. Liu, H. S. Kim, Y.-Y. Noh and D.-H. Hwang, *J. Phys. Chem. C*, 2020, **124**, 20784-20793.
2. R. A. Schlitz, F. G. Brunetti, A. M. Glaudell, P. L. Miller, M. A. Brady, C. J. Takacs, C. J. Hawker and M. L. Chabinyc, *Adv. Mater.*, 2014, **26**, 2825-2830.
3. K. Shi, F. Zhang, C.-A. Di, T.-W. Yan, Y. Zou, X. Zhou, D. Zhu, J.-Y. Wang and J. Pei, *J. Am. Chem. Soc.*, 2015, **137**, 6979-6982.
4. S. Wang, H. Sun, U. Ail, M. Vagin, P. O. Å. Persson, J. W. Andreasen, W. Thiel, M. Berggren, X. Crispin, D. Fazzi and S. Fabiano, *Adv. Mater.*, 2016, **28**, 10764-10771.
5. Y. Wang, M. Nakano, T. Michinobu, Y. Kiyota, T. Mori and K. Takimiya, *Macromolecules*, 2017, **50**, 857-864.
6. D. Kiefer, A. Giovannitti, H. Sun, T. Biskup, A. Hofmann, M. Koopmans, C. Cendra, S. Weber, L. J. Anton Koster, E. Olsson, J. Rivnay, S. Fabiano, I. McCulloch and C. Müller, *ACS Energy Lett.*, 2018, **3**, 278-285.
7. J. Liu, L. Qiu, R. Alessandri, X. Qiu, G. Portale, J. Dong, W. Talsma, G. Ye, A. A. Sengrian, P. C. T. Souza, M. A. Loi, R. C. Chiechi, S. J. Marrink, J. C. Hummelen and L. J. A. Koster, *Adv. Mater.*, 2018, **30**, 1704630.
8. S. Wang, H. Sun, T. Erdmann, G. Wang, D. Fazzi, U. Lappan, Y. Puttisong, Z. Chen, M. Berggren, X. Crispin, A. Kiriy, B. Voit, T. J. Marks, S. Fabiano and A. Facchetti, *Adv. Mater.*, 2018, **30**, 1801898.
9. C.-Y. Yang, W.-L. Jin, J. Wang, Y.-F. Ding, S. Nong, K. Shi, Y. Lu, Y.-Z. Dai, F.-D. Zhuang, T. Lei, C.-A. Di, D. Zhu, J.-Y. Wang and J. Pei, *Adv. Mater.*, 2018, **30**, 1802850.
10. X. Yan, M. Xiong, J.-T. Li, S. Zhang, Z. Ahmad, Y. Lu, Z.-Y. Wang, Z.-F. Yao, J.-Y. Wang, X. Gu and T. Lei, *J. Am. Chem. Soc.*, 2019, **141**, 20215-20221.
11. J. Liu, Y. Shi, J. Dong, M. I. Nugraha, X. Qiu, M. Su, R. C. Chiechi, D. Baran, G. Portale, X. Guo and L. J. A. Koster, *ACS Energy Lett.*, 2019, **4**, 1556-1564.
12. Y. Lu, Z.-D. Yu, R.-Z. Zhang, Z.-F. Yao, H.-Y. You, L. Jiang, H.-I. Un, B.-W. Dong, M. Xiong, J.-Y. Wang and J. Pei, *Angew. Chem. Int. Ed.*, 2019, **58**, 11390-11394.
13. T. L. Dexter Tam, T. T. Lin, M. I. Omer, X. Wang and J. Xu, *J. Mater. Chem. A*, 2020, **8**, 18916-18924.
14. Y. Lu, Z.-D. Yu, Y. Liu, Y.-F. Ding, C.-Y. Yang, Z.-F. Yao, Z.-Y. Wang, H.-Y. You, X.-F. Cheng, B. Tang, J.-Y. Wang and J. Pei, *J. Am. Chem. Soc.*, 2020, **142**, 15340-15348.
15. Y. Wang and K. Takimiya, *Adv. Mater.*, 2020, **32**, 2002060.
16. C. Dong, B. Meng, J. Liu and L. Wang, *ACS Appl. Mater. Interfaces*, 2020, **12**, 10428-

10433.

17. M. Alsufyani, R. K. Hallani, S. Wang, M. Xiao, X. Ji, B. D. Paulsen, K. Xu, H. Bristow, H. Chen, X. Chen, H. Sirringhaus, J. Rivnay, S. Fabiano and I. McCulloch, *J. Mater. Chem. C*, 2020, **8**, 15150-15157.
18. K. Feng, H. Guo, J. Wang, Y. Shi, Z. Wu, M. Su, X. Zhang, J. H. Son, H. Y. Woo and X. Guo, *J. Am. Chem. Soc.*, 2021, **143**, 1539-1552.
19. T. L. D. Tam, M. Lin, A. D. Handoko, T. T. Lin and J. Xu, *J. Mater. Chem. A*, 2021, **9**, 11787-11793.
20. C.-Y. Yang, M.-A. Stoeckel, T.-P. Ruoko, H.-Y. Wu, X. Liu, N. B. Kolhe, Z. Wu, Y. Puttisong, C. Musumeci, M. Massetti, H. Sun, K. Xu, D. Tu, W. M. Chen, H. Y. Woo, M. Fahlman, S. A. Jenekhe, M. Berggren and S. Fabiano, *Nat. Commun.*, 2021, **12**, 2354.
21. J. Liu, G. Ye, H. G. O. Potgieser, M. Koopmans, S. Sami, M. I. Nugraha, D. R. Villalva, H. Sun, J. Dong, X. Yang, X. Qiu, C. Yao, G. Portale, S. Fabiano, T. D. Anthopoulos, D. Baran, R. W. A. Havenith, R. C. Chiechi and L. J. A. Koster, *Adv. Mater.*, 2021, **33**, 2006694.
22. G. Ye, J. Liu, X. Qiu, S. Stäter, L. Qiu, Y. Liu, X. Yang, R. Hildner, L. J. A. Koster and R. C. Chiechi, *Macromolecules*, 2021, **54**, 3886-3896.
23. J. Han, C. Ganley, Q. Hu, X. Zhao, P. Clancy, T. P. Russell and H. E. Katz, *Adv. Funct. Mater.*, 2021, **31**, 2010567.
24. Y. Lu, Z.-D. Yu, H.-I. Un, Z.-F. Yao, H.-Y. You, W. Jin, L. Li, Z.-Y. Wang, B.-W. Dong, S. Barlow, E. Longhi, C.-a. Di, D. Zhu, J.-Y. Wang, C. Silva, S. R. Marder and J. Pei, *Adv. Mater.*, 2021, **33**, 2005946.
25. C. Dong, S. Deng, B. Meng, J. Liu and L. Wang, *Angew. Chem. Int. Ed.*, 2021, **60**, 16184-16190.
26. H. Chen, M. Moser, S. Wang, C. Jellett, K. Thorley, G. T. Harrison, X. Jiao, M. Xiao, B. Purushothaman, M. Alsufyani, H. Bristow, S. De Wolf, N. Gasparini, A. Wadsworth, C. R. McNeill, H. Sirringhaus, S. Fabiano and I. McCulloch, *J. Am. Chem. Soc.*, 2021, **143**, 260-268.
27. A. Marks, X. Chen, R. Wu, R. B. Rashid, W. Jin, B. D. Paulsen, M. Moser, X. Ji, S. Griggs, D. Meli, X. Wu, H. Bristow, J. Strzalka, N. Gasparini, G. Costantini, S. Fabiano, J. Rivnay and I. McCulloch, *J. Am. Chem. Soc.*, 2022, **144**, 4642-4656.
28. Y. Shi, J. Li, H. Sun, Y. Li, Y. Wang, Z. Wu, S. Y. Jeong, H. Y. Woo, S. Fabiano and X. Guo, *Angew. Chem. Int. Ed.*, 2022, **61**, e202214192.
